# Eco-Geographical Diversification of Bitter Taste Receptor Genes (*TAS2R*s) among Subspecies of Chimpanzees (*Pan troglodytes*)

**DOI:** 10.1371/journal.pone.0043277

**Published:** 2012-08-16

**Authors:** Takashi Hayakawa, Tohru Sugawara, Yasuhiro Go, Toshifumi Udono, Hirohisa Hirai, Hiroo Imai

**Affiliations:** 1 Molecular Biology Section, Department of Cellular and Molecular Biology, Primate Research Institute, Kyoto University, Inuyama, Aichi, Japan; 2 Japan Society for Promotion of Science, Tokyo, Japan; 3 Kumamoto Sanctuary, Wildlife Research Center of Kyoto University, Uki, Kumamoto, Japan; German Institute for Human Nutrition, Germany

## Abstract

Chimpanzees (*Pan troglodytes*) have region-specific difference in dietary repertoires from East to West across tropical Africa. Such differences may result from different genetic backgrounds in addition to cultural variations. We analyzed the sequences of all bitter taste receptor genes (c*TAS2R*s) in a total of 59 chimpanzees, including 4 putative subspecies. We identified genetic variations including single-nucleotide variations (SNVs), insertions and deletions (indels), gene-conversion variations, and copy-number variations (CNVs) in c*TAS2R*s. Approximately two-thirds of all c*TAS2R* haplotypes in the amino acid sequence were unique to each subspecies. We analyzed the evolutionary backgrounds of natural selection behind such diversification. Our previous study concluded that diversification of c*TAS2R*s in western chimpanzees (*P. t. verus*) may have resulted from balancing selection. In contrast, the present study found that purifying selection dominates as the evolutionary form of diversification of the so-called human cluster of c*TAS2R*s in eastern chimpanzees (*P. t. schweinfurthii*) and that the other c*TAS2R*s were under no obvious selection as a whole. Such marked diversification of c*TAS2R*s with different evolutionary backgrounds among subspecies of chimpanzees probably reflects their subspecies-specific dietary repertoires.

## Introduction

One genus of the great apes living in areas spanning from East to West across tropical Africa is *Pan*, which commonly consists of 2 species, chimpanzees (*Pan troglodytes*) and bonobos (*Pan paniscus*). Chimpanzees are also divided into 4 subspecies: eastern chimpanzees (*Pan troglodytes schweinfurthii*), central chimpanzees (*Pan troglodytes troglodytes*), Nigerian-Cameroonian chimpanzees (*Pan troglodytes ellioti* or formerly *Pan troglodytes vellerosus*), and western chimpanzees (*Pan troglodytes verus*), defined by their geographical ranges [1]–[3]. These 4 subspecies are genetically distinguishable from one another by their mitochondrial DNA (mtDNA) sequences [1], [4]. Ecological differences among these subspecies have also been described. Long-term field studies have pointed to the variability of behavioral and dietary repertoires of chimpanzees among study sites from East to West across tropical Africa, suggesting significant “cultural” differentiations both within and among subspecies [5]–[7]. Molecular ecologists have shown that such “cultural” dissimilarity in behavior among chimpanzees is correlated with genetic differences of mtDNA D-loop sequences across the geographical range [8]. However, intergroup differences in chimpanzee behavior have not been sufficiently discussed from the viewpoint of genetic diversification of functional genes.

**Figure 1:**
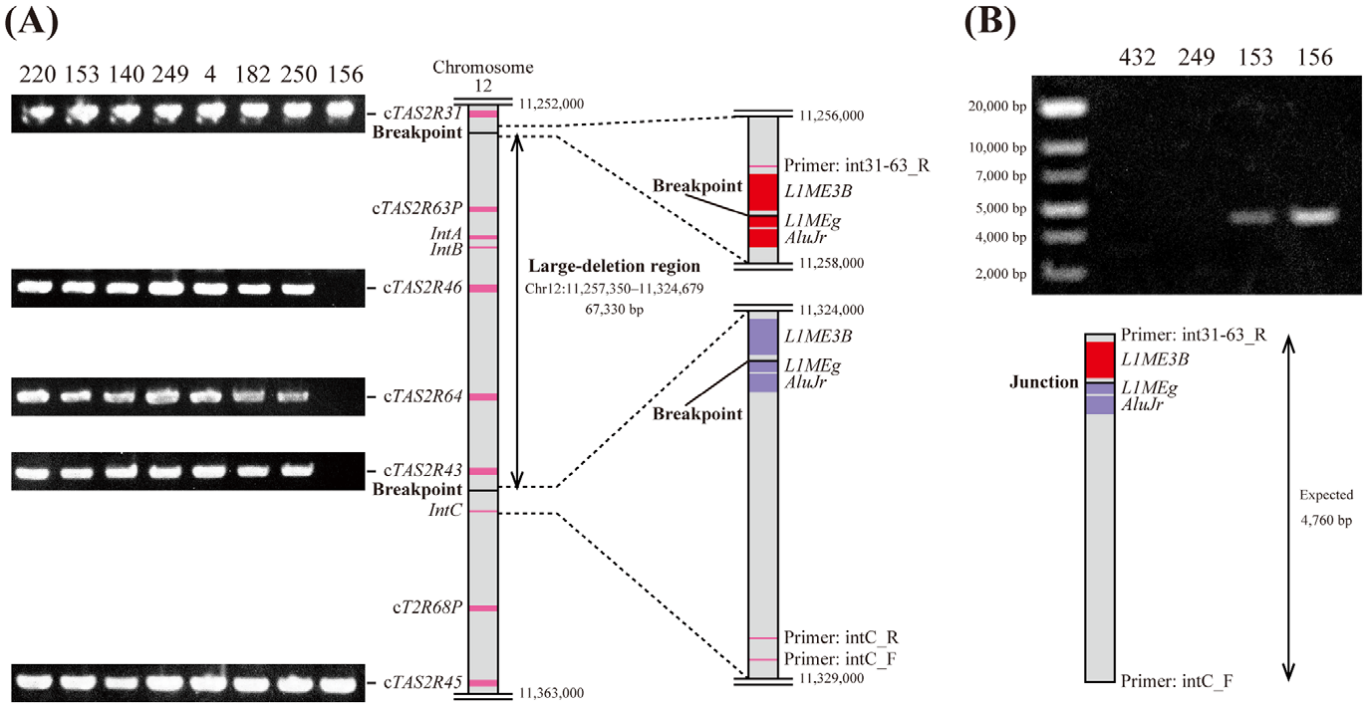
A large-deletion variant involving the whole-gene deletions of c*TAS2R43*, c*TAS2R46*, c*TAS2R63P*, and c*TAS2R64*. (A) Genomic organization around the large-deletion region based on CGSC 2.1.3/panTro3. An electrophoresis image shows PCR products of each c*TAS2R* with subject ID numbers at the top. Only subject 156 did not produce amplicons of c*TAS2R43*, c*TAS2R46*, and c*TAS2R64*. In this subject, c*TAS2R63P*, *IntA*, and *IntB* were also not amplified, whereas *IntC* was amplified (data not shown). (B) Using intC_F and int31-63_R as a PCR primer pair, only subject 153 and 156 produced amplicons of the expected size of 4,760 bp based on CGSC 2.1.3/panTro3. Subjects 153 and 156 were thought to be a heterozygote and a homozygote for the large-deletion variant, respectively. The sequences around the breakpoints of the large-deletion variant had similar arrangements of retrotransposons (*AluJr*, *L1MEg*, and *L1ME3B*), which were annotated with RepeatMasker (http://www.repeatmasker.org).

Taste allows mammals to evaluate their foods and determine which foods they can ingest. Thus, it is one of the pivotal senses for mammals. Among several kinds of taste, bitter taste has been evolutionarily highly developed, because bitter compounds, which are usually poisonous, are more variable than nutrients such as sweet or umami compounds. In mammals, bitter taste is mediated by several dozen G-protein-coupled taste 2 receptors (bitter taste receptors, T2Rs, TAS2Rs), whereas sweet or umami taste is mediated by only a few G-protein-coupled taste 1 receptors (sweet or umami taste receptors, T1Rs, TAS1Rs). This difference in abundance suggests that there has been more rapid adaptive evolution of bitter taste receptors to variable poisonous compounds in mammalian feeding environments [9]–[14].

Our previous study indicated that western chimpanzee *TAS2R*s have been diversified under balancing selection, probably to recognize a broader range of substances [15]. However, *TAS2R* repertoires and their variations in other subspecies of chimpanzees remain unknown. Wild chimpanzees are omnivores ingesting more than 200 plant species and many types of insects and vertebrates [6], [16]–[19]. Regional differences in dietary repertoires among chimpanzees also include several types of bitter-tasting plants [6], [16], [20]. Since the relationship between behavioral and genetic variation is not yet clear, we intended to determine whether such differences in usage of bitter-tasting plants result from genetic diversity of bitter taste receptors. In this study, we accordingly characterized patterns of intrasubspecific nucleotide diversity and intersubspecific nucleotide divergence of chimpanzee *TAS2R*s (c*TAS2R*s) sequenced from all putative subspecies of chimpanzees, and identified signatures of natural selection on c*TAS2R*s, which implies the significance of the genetic background on the dietary repertoires of chimpanzees.

**Table 1:** Variations of loss of start codon, gain of stop codon, indel, gene conversion, and whole-gene deletion in c*TAS2R*s.

			Allele frequency			
c*TAS2R*	Mutation^a^	Function	Western	Eastern	Central	Nigerian-Cameroonian^b^
c*TAS2R1*	1 bp del.	Non-functional	22/92 (24%)	0	0	0
c*TAS2R3*	LIC^c^	Functional	75/92 (82%)	20/20 (100%)	4/4 (100%)	2/2 (100%)
c*TAS2R7*	GTC	Non-functional	0	0	1/4 (25%)	0
c*TAS2R30*	19 bp ins.	Non-functional	3/92 (3%)	0	0	0
c*TAS2R31*	GTC	Non-functional	9/92 (10%)	0	0	0
c*TAS2R38*	LIC	Non-functional	70/92 (76%)	0	0	0
c*TAS2R40*	GTC	Non-functional	0	1/20 (5%)	0	0
c*TAS2R42*	5 bp del.	Non-functional	24/92 (26%)	10/20 (50%)	1/4 (25%)	1/2 (50%)
c*TAS2R43*	1 bp ins.	Non-functional	11/92 (12%)	0	0	0
c*TAS2R43*	LIC	Non-functional	0	3/20 (15%)	0	0
c*TAS2R43*	WGD	Non-functional	0	3/20 (15%)	0	0
c*TAS2R45*	4 bp del.	Non-functional	11/92 (12%)	0	0	0
c*TAS2R45*	LIC	Non-functional	0	0	3/4 (75%)	0
c*TAS2R46*	GTC	Non-functional	0	3/20 (15%)	0	0
c*TAS2R46*	WGD	Non-functional	0	3/20 (15%)	0	0
c*TAS2R46*	73 bp GC^d^	Functional	0	0	0	1/2 (50%)
c*TAS2R60*	2 bp del.	Non-functional	0	0	2/4 (50%)	1/2 (50%)
c*TAS2R64*	WGD	Non-functional	0	3/20 (15%)	0	0
c*TAS2R64*	133 bp GC^e^	Functional	0	2/20 (10%)	0	0

a Abbreviations: del., deletion; ins., insertion; LIC, loss of start (initiation) codon; GTC, gain of stop (termination) codon; WGD, whole-gene deletion; GC, gene conversion. There was no variation of loss of stop codon in c*TAS2R*s.

b The subspecies of the individual was identified only maternally due to the lack of information about the antecedents in captivity.

c The alleles have an alternative start codon at nucleotide positions 4 to 6, and thus are expected to be functional.

d Gene conversion with c*TAS2R31* at nucleotide positions 514 to 586, expected to be functional.

e Gene conversion with c*TAS2R19* at nucleotide positions 141 to 273, expected to be functional.

**Figure 2:**
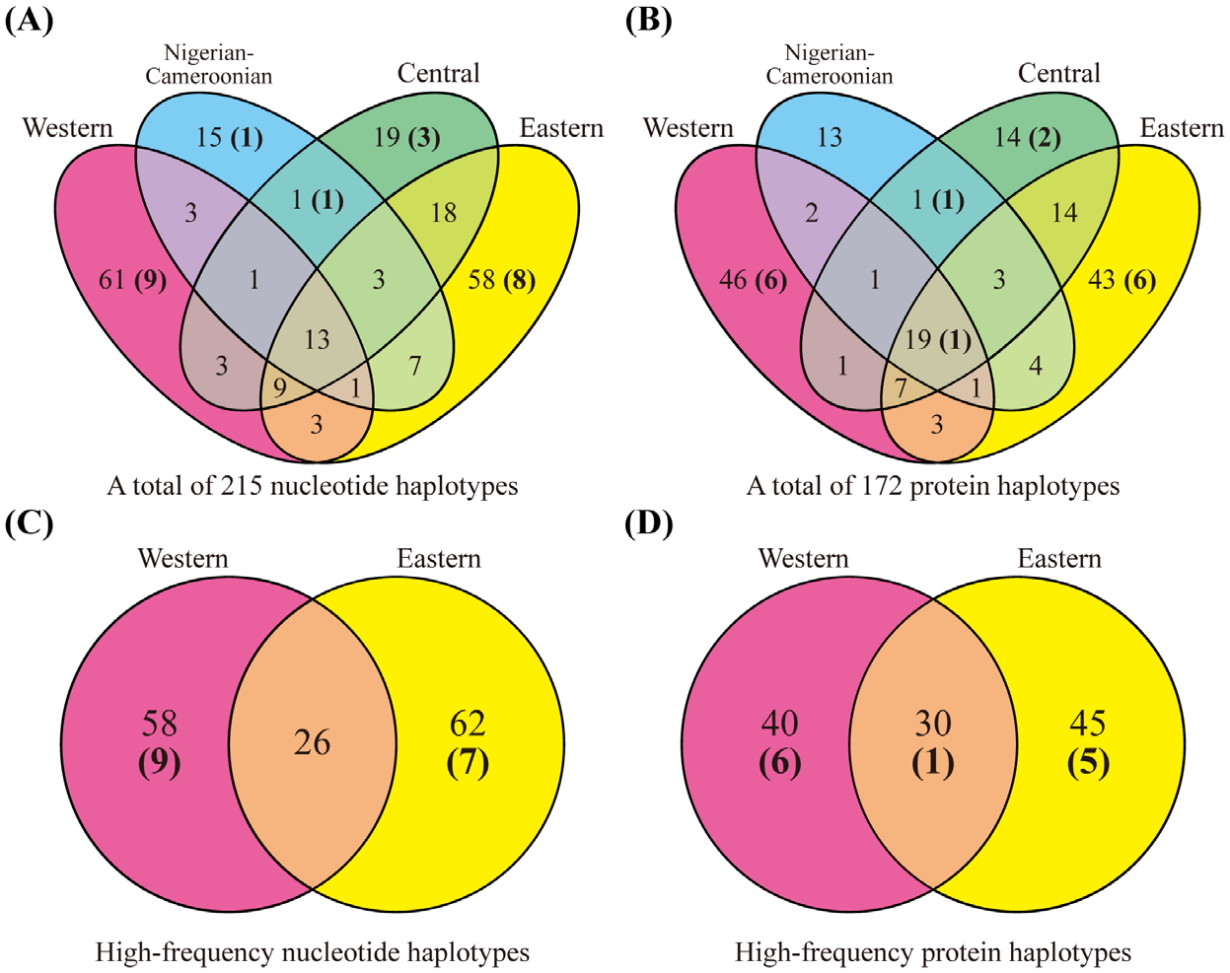
The subspecies distribution of the number of haplotypes in the 28 c*TAS2R*s. (A, B) The distribution of all haplotypes in the 4 subspecies. (C, D) The distribution of high-frequency haplotypes in western and eastern chimpanzees. Subspecies of a Nigerian-Cameroonian chimpanzee was identified only maternally due to a lack of information about the antecedents in captivity. The number of non-functional haplotypes (segregating pseudogenes and whole-gene deletions) is indicated in parentheses. High-frequency haplotypes were observed in more than one sampled chromosome.

## Results

### Nucleotide Variations in c*TAS2R*s among the Subspecies of Chimpanzees

We determined the sequence variations in all 28 c*TAS2R*s from 59 subjects (10 eastern and 2 central chimpanzees, a putative Nigerian-Cameroonian chimpanzee, and 46 western chimpanzees previously analyzed). Of the 28 c*TAS2R*s, 3 genes tandemly located on chromosome 12 (c*TAS2R43*, c*TAS2R46*, and c*TAS2R64*) could not be amplified by polymerase chain reaction (PCR) from an eastern chimpanzee (Figure 1A). Speculating that this result was due to a large deletion involving these tandem c*TAS2R*s, we attempted to amplify and sequence the neighboring regions of these c*TAS2R*s. We confirmed the presence of a large-deletion variant resulting in whole-gene deletions of these c*TAS2R*s and a pseudogene of c*TAS2R63P* in this subject as a homozygote (Figure 1B). Another eastern chimpanzee also carried the large-deletion variant as a heterozygote. We identified the breakpoints of the large-deletion region using BLAT [21]. The breakpoint sequence is shown in Figure S1. The span between the breakpoints was estimated to be 67,330 bp. Since the sequences around the breakpoints have similar arrangements of retrotransposons, the large-deletion variant was possibly produced by ectopic homologous recombination as a result of hybridization of these tandem homologous sequences. Thus, it was found that eastern chimpanzees have copy-number variations (CNVs) in c*TAS2R43*, c*TAS2R46*, and c*TAS2R64*.

**Figure 3:**
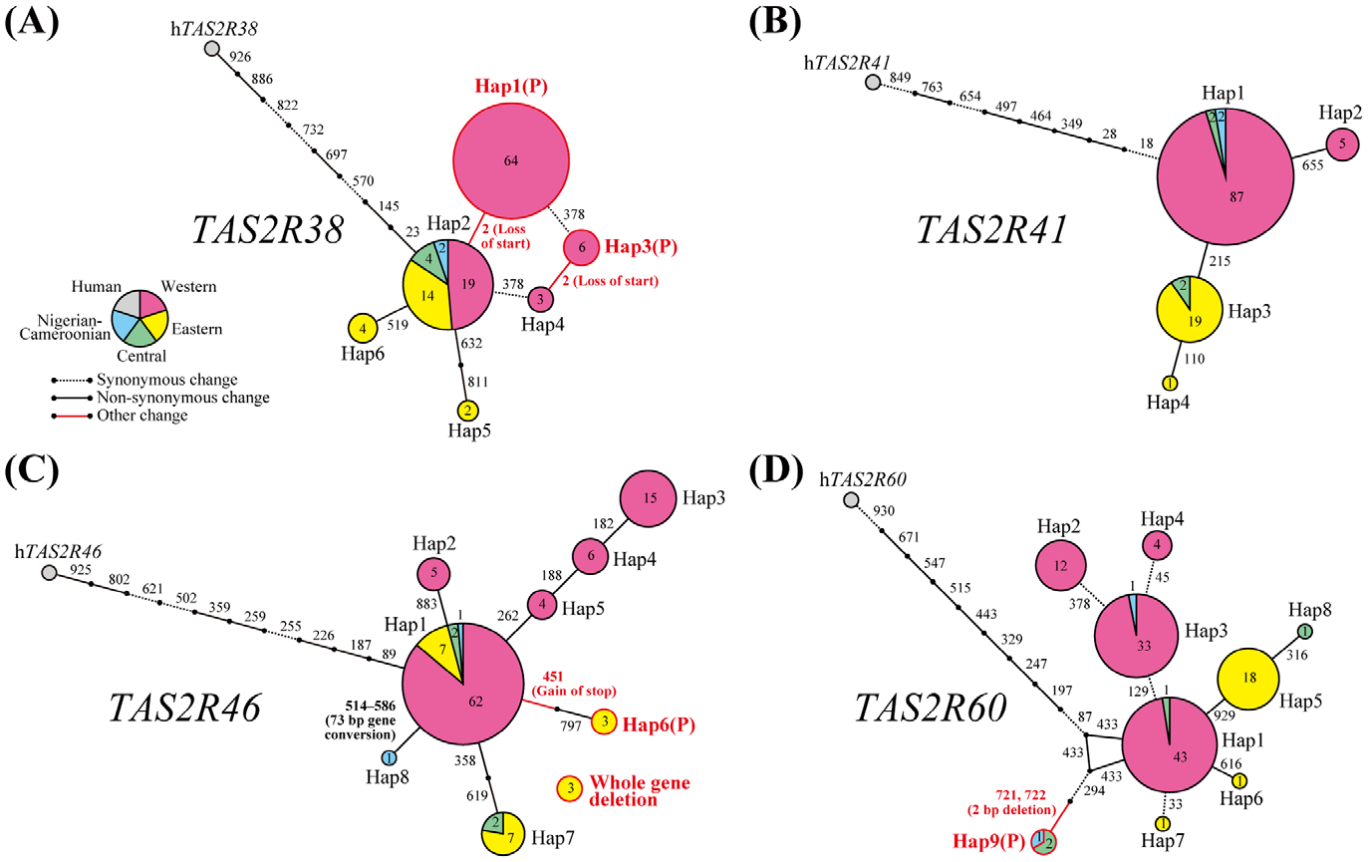
Median-joining networks for c*TAS2R*s. Circles represent haplotypes. Hap*n* indicates haplotype *n* (Table S1). The letter (P) is added to pseudogenes. Color within the circle indicates each subspecies. Areas and numbers within color-coded parts of the circles indicate the numbers of sampled chromosomes. Numbers along branches indicate nucleotide positions of mutation between the haplotypes. Line styles of branches indicate mutation types of nucleotide changes.

**Table 2:** Nucleotide diversity and divergence of c*TAS2R*s in western and eastern chimpanzees.

				Western chimpanzee^a^				Eastern chimpanzee				Between
Class	Locus	Chromosome	Length	*π* b	*π*_S_ c	*π*_N_ d	*D* e	*π* b	*π*_S_ c	*π*_N_ d	*D* e	*d*_XY_ f
Old c*TAS2R*s	c*TAS2R1*	5	900	0.043	0.000	0.057	−0.012	0.244	0.460	0.181	0.369	0.211
	c*TAS2R2*	7	912	0.096	0.200	0.067	1.904	0.069	0.000	0.089	0.282	0.101
	c*TAS2R3*	7	951	0.141	0.000	0.144	1.413	0.071	0.044	0.081	−0.526	0.138
	c*TAS2R4*	7	900	0.056	0.000	0.075	1.835	0.060	0.201	0.015	−0.090	0.096
	c*TAS2R5*	7	900	0.016	0.000	0.021	−0.322	0.000	0.000	0.000	NA	0.008
	c*TAS2R7*	12	978	0.025	0.109	0.000	0.291	0.295	0.197	0.329	0.478	0.214
	c*TAS2R8*	12	930	0.052	0.191	0.012	−0.320	0.068	0.000	0.088	0.282	0.077
	c*TAS2R9*	12	939	0.043	0.000	0.056	1.211	0.095	0.000	0.125	−0.598	0.093
	c*TAS2R10*	12	924	0.002	0.000	0.003	−1.037	0.071	0.049	0.078	−1.206	0.039
	c*TAS2R13*	12	912	0.063	0.252	0.006	−0.903	0.011	0.048	0.000	−1.164	0.040
	c*TAS2R14*	12	954	0.072	0.000	0.095	0.317	0.066	0.000	0.086	0.282	0.092
	c*TAS2R16*	7	876	0.031	0.000	0.041	0.473	0.091	0.000	0.119	−1.334	0.070
	c*TAS2R38*	7	1002	0.055	0.074	0.000	0.612	0.071	0.140	0.050	−0.410	0.126
	c*TAS2R39*	7	1017	0.024	0.000	0.032	0.291	0.057	0.000	0.075	0.085	0.048
	c*TAS2R40*	7	972	0.000	0.000	0.000	NA	0.045	0.147	0.000	−0.528	0.026
	c*TAS2R41*	7	924	0.011	0.000	0.015	−0.549	0.011	0.000	0.014	−1.164	0.120
	c*TAS2R42*	12	945	0.207	0.325	0.174	1.489	0.235	0.542	0.146	1.739	0.254
	c*TAS2R60* (c*T2R56*)	7	957	0.086	0.357	0.000	0.705	0.041	0.044	0.040	−1.441	0.179
	c*TAS2R62*	7	939	0.132	0.184	0.117	1.168	0.117	0.083	0.129	−0.683	0.203
	Concatenated	–	17832	0.061	0.089	0.048	–	0.091	0.103	0.087	–	0.113
Human cluster	c*TAS2R19* (c*T2R48*)	12	900	0.022	0.000	0.029	−0.003	0.191	0.314	0.155	0.677	0.140
	c*TAS2R20* (c*T2R49*)	12	930	0.117	0.000	0.154	1.555	0.076	0.000	0.099	−0.443	0.154
	c*TAS2R30* (c*T2R47*)	12	960	0.053	0.000	0.069	−0.717	0.206	0.485	0.124	−0.413	0.146
	c*TAS2R31* (c*T2R44*)	12	930	0.091	0.216	0.030	0.807	0.151	0.374	0.085	−0.020	0.136
	c*TAS2R43*	12	930	0.197	0.204	0.197	2.689*	0.342	0.703	0.198	−0.088	0.324
	c*TAS2R45*	12	930	0.117	0.100	0.124	1.535	0.130	0.347	0.066	−1.279	0.151
	c*TAS2R46*	12	930	0.122	0.000	0.160	0.899	0.177	0.000	0.188	1.200	0.204
	c*TAS2R50*	12	900	0.237	0.413	0.187	1.316	0.111	0.253	0.070	1.814	0.200
	c*TAS2R64*	12	930	0.074	0.000	0.098	0.302	0.137	0.000	0.181	0.120	0.138
	Concatenated	–	8340	0.114	0.101	0.116	–	0.169	0.273	0.130	–	0.177

a Data from Sugawara et al. [15].

b Nucleotide diversity within the subspecies.

c Synonymous diversity within the subspecies.

d Non-synonymous diversity within the subspecies.

e Tajima’s *D*. Two-sided Tajima’s *D* test were conducted using coalescent simulations under 10,000 replicates, assuming no recombination and a Poisson distribution of mutations along the lineages. *P<0.05.

f Nucleotide divergence beween western and eastern chimpanzees.

A total of 215 nucleotide and 172 protein haplotypes were identified in the 28 c*TAS2R*s. The genotype of each subject is summarized in Table S1. Of the haplotypes, 16 protein haplotypes are expected to be non-functional (segregating pseudogenes and whole-gene deletions) and 2 are expected to be produced by ectopic gene conversion (Table 1). The haplotype distribution in the 4 subspecies of chimpanzees is shown in Figure 2A, 2B and Table S2. Approximately two-thirds of all the protein haplotypes (116/172) were unique to each subspecies, suggesting marked diversification of c*TAS2R*s at the subspecies level. Since it is possible that rare but potentially shared haplotypes are less likely to be found in more than one subspecies if the sampling number is limited, we also depicted distribution of high-frequency haplotypes in western and eastern chimpanzees (Figure 2C, 2D). As a result, the frequency of subspecies-specific protein haplotypes was largely retained without being influenced by rare haplotypes (114/144 to 85/115 in western and eastern chimpanzees). Furthermore, we conducted Monte Carlo simulations to reconstruct the haplotype distribution in western and eastern chimpanzees (Figure S2). Assuming no differentiation in the 2 populations, the mean total number of shared haplotypes would be expected to be 125.1 (nucleotide) and 101.8 (protein) under 10,000 replicates of the simulation. The observed total number of shared haplotypes (26 and 30, respectively) is far below these expected values. Therefore, numerous subspecies-specific haplotypes are significantly distributed in western and eastern chimpanzees by genetic drift and/or natural selection rather than experimental sampling bias (*P*<0.0001). In addition, despite a few samples from Nigerian-Cameroonian and central chimpanzees, unique haplotypes were frequently observed in these subspecies. Because rare haplotypes are less likely to be observed from a few samples, this result indicates that c*TAS2R*s of Nigerian-Cameroonian and central chimpanzees would be differentiated from those of western and eastern chimpanzees.

### Natural Selection of c*TAS2R*s in Western and Eastern Chimpanzees

**Figure 4:**
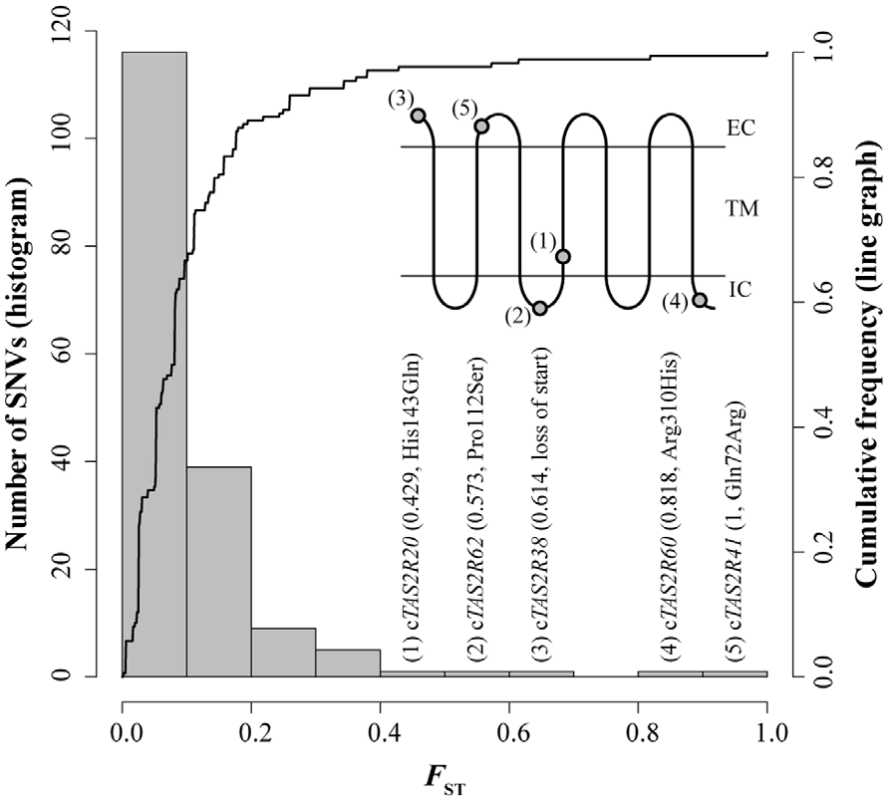
The histogram and cumulative frequency curve of *F*_ST_ in SNVs in the 28 c*TAS2R*s. This plot was composed from a total of 174 SNVs in western and eastern chimpanzees. Sampled chromosomes carrying whole-gene deletions were omitted from the calculation. Mutation types and amino acid positions of SNVs with higher *F*_ST_ are shown. Ancestral and derived amino acids were estimated from the haplotype networks. The protein locations are also shown based on Sugawara et al. [15] (EC, extracellular region; TM, transmembrane region; IC, intracellular region).

We constructed haplotype networks for each c*TAS2R* with the corresponding human *TAS2R* (h*TAS2R*). Some representative networks are shown in Figure 3. Most networks were composed of a few haplotypes shared among subspecies and many derived haplotypes unique to each subspecies (Figure 3A, 3C). On the other hand, some networks did not show protein haplotypes shared in both western and eastern chimpanzees (Figure 3B, 3D). To evaluate the level of divergence among single-nucleotide variations (SNVs), we calculated *F*_ST_ for each of the 174 SNVs in all c*TAS2R*s between western and eastern chimpanzees (Figure 4). We also reanalyzed the distribution of *F*_ST_ in 194 SNVs in a total of 22,401 bp of 26 putatively neutral, non-coding loci in 10 western and 10 eastern chimpanzees reported by Fischer et al. [22]. The *F*_ST_ distribution of the non-coding SNVs provided an *F*_ST_ of more than 0.6 with less than 5% empirical probability. In the SNVs of c*TAS2R*s, Gln72Arg of c*TAS2R41* (*F*_ST_ = 1), Arg310His of c*TAS2R60* (*F*_ST_ = 0.818), and a loss-of-start SNV of c*TAS2R38* (*F*_ST_ = 0.614) showed *F*_ST_ of more than 0.6. Derived alleles of the non-synonymous SNVs in c*TAS2R41* and c*TAS2R60* were present only in eastern chimpanzees (Figure 3B, 3D), whereas derived alleles of the loss-of-start SNV in c*TAS2R38* were present only in western chimpanzees (Figure 3A). The divergence of these SNVs was inconsistent with selective neutrality between western and eastern chimpanzees.

We also examined the trend of natural selection in c*TAS2R*s. Primate *TAS2R*s are classified into 2 classes (the human cluster and other phylogenetically old *TAS2R*s) [23]. *TAS2R*s in the human cluster are thought to be sub-functionalized as a result of recent gene duplications in the primate lineage and to be under different selective constraints from the other old *TAS2R*s. We accordingly analyzed these 2 classes. We estimated nucleotide diversity (*π*) and divergence (*d*_XY_) of c*TAS2R*s in western and eastern chimpanzees (Table 2). In concatenated sequences of both classes, the *π* value of western chimpanzees was lower than that of eastern chimpanzees and the *d*_XY_ value, whereas the *π* value of eastern chimpanzees did not differ greatly from the *d*_XY_ value. This observation indicates that eastern chimpanzees have higher heterozygosity and more ancestral SNVs in c*TAS2R*s than western chimpanzees. The *π* and *d*_XY_ values in the 26 non-coding loci reported by Fischer et al. [22] showed a similar tendency to those in c*TAS2R*s. This tendency of diversity and divergence of c*TAS2R*s is expected to reflect the demographic history of chimpanzee subspecies.

Our previous study revealed that Tajima’s *D* distribution of the 28 c*TAS2R*s of western chimpanzees was significantly higher than that of non-coding loci of western chimpanzees, suggesting balancing selection as the general form of natural selection [15]. This form was also observed after classification into the human cluster and the old c*TAS2R*s (Table 3). In contrast, in comparison of Tajima’s *D* distribution of eastern chimpanzee *TAS2R*s with that of the 26 non-coding loci of eastern chimpanzees reported by Fischer et al. [22], there was no significant difference in both classes (Table 3). To examine the selective constraints on amino acid sequences, we also estimated the synonymous (*π*_S_) and non-synonymous diversity (*π*_N_) in each class and subspecies. In the concatenated sequence, only the *π*_N_/*π*_S_ value of the human cluster in eastern chimpanzees was significantly less than 1 following Zhang et al. [24] (Table 3). This implies that purifying selection is the dominant evolutionary form of diversification of eastern chimpanzee *TAS2R*s in the human cluster. These results suggest that the haplotype repertoires of western and eastern chimpanzee *TAS2R*s have been formed by different patterns of natural selection.

## Discussion

Most of the 215 nucleotide and 172 protein haplotypes of c*TAS2R*s were specific to each subspecies (Figure 2), suggesting the subspecies specificity for the sense of bitter taste, if these haplotype differences are assumed to be associated with functional differences. Although numerous protein haplotypes of h*TAS2R*s as well as c*TAS2R*s were reported (e.g., a total of 151 nucleotide haplotypes observed in 25 h*TAS2R*s in the global population [25]), only a few functional differences among these haplotypes, particularly those related to non-synonymous variations, have been documented until date. For example, 6 intact protein haplotypes of h*TAS2R31* were observed in Caucasians and their protein functions were investigated by further cell-based assays [26]. Of them, one haplotype was highly responsive to saccharin and acesulfame K but not the others. This result indicates that not all differences in protein haplotypes are linked to functional differences. More than half of the amino acids involved in these h*TAS2R31* haplotype differences interacted with each other for the function. Therefore, functional similarity of the less responsiveness does not necessarily result from common mechanism. There are also several non-synonymous variations changing their protein functions in h*TAS2R9*, h*TAS2R16*, h*TAS2R38*, and h*TAS2R43*, as reported by cell-based assays [26]–[30]. Furthermore, a derived protein haplotype of c*TAS2R16*, which was revealed to be specific to western chimpanzees in this study (Table S2), shows approximately only half the sensitivity of the ancestral haplotype to β-glucopyranosides in cell-based assays [15], [31]. Variations of pseudogenes and whole-gene deletions could drastically affect the phenotypes. It was revealed that a whole-gene deletion in h*TAS2R43* results in psychologically less sensitivity to the specific bitter compounds [26], [30]. A segregating pseudogene in c*TAS2R38* also results in the less sensitivity [32]. We found that this pseudogenized c*TAS2R38* was specific to western chimpanzees, whose strong divergence was significantly supported by *F*_ST_ analysis (see below regarding evolutionary interpretation). Phenotype differences would also be influenced by other pseudogenes and whole-gene deletions specific to each subspecies. While the function of each protein haplotype in c*TAS2R*s should be investigated in future, some of the subspecies-specific protein haplotypes in c*TAS2R*s may potentially relate to functional differences, and presence of the subspecies specificity for the sense of bitter taste could be indicated.

The evolutionary forms of natural selection of c*TAS2R*s were also different between western and eastern chimpanzees (Table 3). The evolution of eastern chimpanzee *TAS2R*s within the human cluster was characterized by purifying selection, in marked contrast to the balancing selection in western chimpanzees. Since the early gene expansion of some *TAS2R*s of the human cluster may have occurred under positive selection in the primate lineage [23], the current sequence stability of those genes by purifying selection after early functional divergence may be more important than the other old c*TAS2R*s in eastern chimpanzees. On the other hand, the occurrence of balancing selection in western chimpanzees may be due to demographic factors. Sugawara et al. [15] hypothesized that balancing selection in western chimpanzees had occurred to favor the increase in the number of individuals heterozygous for functionally divergent alleles. Even if such a form of evolution is shared by eastern chimpanzees, they already have higher genome-wide heterozygosity than western chimpanzees due to demographic factors [22], and thus, balancing selection may not have been required in eastern chimpanzees. These different patterns of natural selection would contribute to the formation of different haplotype repertoires of c*TAS2R*s at the subspecies level.

Non-synonymous SNVs in c*TAS2R41* and c*TAS2R60* and a loss-of-start SNV in c*TAS2R38* were significantly different between western and eastern chimpanzees with higher *F*_ST_ (Figure 4). However, the known orthologues of *TAS2R41* and *TAS2R60* are orphans [33], and thus, we cannot discuss them with their ligands in this study. The loss-of-start SNV of c*TAS2R38* is unique to western chimpanzees (Figure 3A), suggesting that the loss-of-start allele appeared and began to spread at around the time of chimpanzee subspeciation, about 0.5 million years ago (mya) [34]–[36]. Wooding et al. [32] found that the loss-of-start variant of c*TAS2R38* confers recessively inherited inability to detect the bitterness of an artificial compound, phenylthiocarbamide (PTC), *in vitro* and *in vivo*. This phenotypic variation of PTC perception has been found in many primate taxa, and the causal gene was first identified as *TAS2R38* in humans [37]–[40]. Different types of nonsense mutations in *TAS2R38* orthologues and PTC inactivation in homozygous individuals were also found in Japanese macaques (*Macaca fuscata*) as types of intraspecific variation [41]. Similar to the occurrence of the non-functional allele of *TAS2R38* about 0.5 mya in chimpanzees, phylogenetic analyses revealed that the non-functional allele of *TAS2R38* arouse between 0.3–1.6 mya in humans and at around 0.5 mya in Japanese macaques [41], [42]. The occurrence of PTC non-tasters in multiple species may thus have resulted from similar selective pressure by a global alteration of climate and flora during the Pleistocene epoch, which was characterized by repeated global glaciations.

In addition to the pseudogenization of c*TAS2R38* in western chimpanzees, we found non-functionalization specific to eastern chimpanzees with high frequency (a total of 30%) in c*TAS2R46* by 2 independent mutation events–pseudogenization and whole-gene deletion (Figure 3C). During the study of the tasting of plant foods by eastern chimpanzees in Mahale, Tanzania, Nishida et al. [20] recorded several bitter plant foods including the pith of a medicinal plant, *Vernonia amygdalina* (Asteraceae). Eastern chimpanzees in Gombe, Tanzania and Kahuzi-Biega, Congo-Kinshasa also ingest the pith of *Vernonia* species [16], [20], [43]. In contrast, western chimpanzees in Bossou, Guinea do not ingest *Vernonia* species despite its presence in the vegetation [6]. *V. amygdalina* contains some sesquiterpene lactones as bioactive (e.g., antiparasitic), bitter compounds [44], [45]. Since hTAS2R46 recognizes many sesquiterpene lactones as specific ligands [46], ancestral cTAS2R46 is also expected to recognize the bitterness of *V. amygdalina*. Therefore, non-functional alleles of c*TAS2R46* in eastern chimpanzees may have reduced the perception of aversive bitterness of *V. amygdalina*, and thus, driven its utilization by this subspecies. Of course, it is possible that other c*TAS2R*s are responsive to compounds contained by *V. amygdalina*, and therefore, such reduction of aversive bitterness may be slight or insignificant. In fact, hTAS2R10 and hTAS2R14 as well as hTAS2R46 are broadly tuned to numerous and various compounds and seem to be activated by some sesquiterpene lactones [33], [46]. In addition, Huffman and Seifu [47] hypothesized that the eastern chimpanzees in Mahale utilize the pith of *V. amygdalina* to control their own illness, in which case, intact c*TAS2R46* would be beneficial to identify the pharmacologically functional bitterness, although it is unknown whether chimpanzees could begin to prefer bitter foods depending on the condition. In contrast, Nishida [48] reported that many apparently healthy chimpanzees in Mahale ingest the pith and leaves of *V. amygdalina* and that the young pith of *V. amygdalina* contains many proteins, suggesting that the ingestion is also interpretable as nutritional purpose. If the nutritional purpose is true, non-functionalized c*TAS2R46* alleles could be beneficial in accepting ingestion of *V. amygdalina*. Whether natural selection on c*TAS2R46* has been exerted by *Vernonia* species is unknown; however, the relationship between allelic diversity of c*TAS2R46* and consumption of *Vernonia* species in eastern chimpanzees are very interesting and can be considered in eco-geographical insights of chimpanzees.

**Table 3:** Analyses of natural selection of c*TAS2R*s in western and eastern chimpanzees.

Subspecies	Class	*π*_N_ */π*_S_	*P* value^a^	Median Tajima’s *D*	*P* value^b^
Western^c^	Old c*TAS2R*s	0.539	0.19	0.395	<0.01
	Human cluster	1.147	0.52	0.899	<0.01
Eastern	Old c*TAS2R*s	0.848	0.65	−0.468	0.61
	Human cluster	0.475	<0.01	−0.020	0.31

a Two-sided Fisher’s exact test.

b Two-sided Wilcoxon rank sum test in comparison with data of non-coding loci from Fischer et al. [22].

c Data from Sugawara et al. [15].

We demonstrated distinct differences in c*TAS2R* haplotype repertoires among the 4 subspecies of chimpanzees. A detailed examination of natural selection revealed different evolutionary backgrounds behind the different haplotype repertoires of western and eastern chimpanzees. Each subspecies of chimpanzees has evolutionarily formed a unique haplotype repertoire of c*TAS2R*s depending on specific demographic and environmental factors. These findings will allow us to clarify the genetic backgrounds of regional dietary repertoires of chimpanzees by further phenotypic analyses of each c*TAS2R* haplotype.

## Materials and Methods

### Ethics Statement

All experiments were performed based on the Guidelines for Care and Use of Nonhuman Primates Version 2 and 3 of the Primate Research Institute, Kyoto University (2002, 2010). The experiments were approved by the Animal Welfare and Animal Care Committee (Monkey Committee) of the Primate Research Institute (2009-1809, 2010-054, 2011-098). All samples (hair and blood) were collected from living chimpanzees by the veterinaries and zookeepers. The hair samples were collected for the purpose of the present study. For well-habituated chimpanzees, the hairs were pulled out by gloved hands. For the other chimpanzee, the fallen hairs in each cage for sleeping were gathered. To minimize suffering, the blood samples were not collected for the purpose of the present study but as part of routine health examinations. The chimpanzees were healthily kept for the purpose of zoological research in the Kumamoto Sanctuary, Wildlife Research Center of Kyoto University and for the purpose of public exhibitions in Japanese zoos in the enough size of enclosure (10 m wide, 10 m depth, and more than 5 m height). Their environments were enriched. For example, they were always provided a variety of foods (vegetables, fruits, and potatoes) scattered in the enclosure and lived together with several chimpanzees of all ages and sexes to satisfy their activity and sociality.

### Samples

Genomic DNA of a total of 13 captive chimpanzees in zoos and research facilities in Japan were analyzed (Table S3). Their maternal origins were identified based on partial mtDNA D-loop sequences, referring to previous literature (e.g., Morin et al. [4]). From their biographical data, all were presumably wild-born chimpanzees, except one whose birthplace is unknown because she was imported from a European zoo. Thus, they comprised 10 eastern and 2 central chimpanzees and an individual about whom it is known that at least her maternal antecedents included Nigerian-Cameroonian chimpanzees. We included the data of 46 western chimpanzees previously reported in Sugawara et al. [15] in the analyses.

### Sequencing and Haplotype Inference of c*TAS2R*s

The chimpanzee genome has 28 presumably intact c*TAS2R*s [15], whose entire coding regions were selected as target loci for PCR amplification and sequencing in this study. Primers were used as shown in Table S4 in addition to ones designed by Sugawara et al. [15]. The PCR mixtures of 25 µl contained ExTaq DNA polymerase (0.625 U) (Takara Bio Inc., Shiga, Japan), the reaction buffer and deoxynucleoside triphosphates (0.2 mM each) provided by the DNA polymerase’s manufacturer, primers (0.2 µM each), and an adequate amount of genomic DNA as the template. PCR was performed with an initial denaturation at 94°C for 10 minutes and 35–40 thermal cycles of denaturation at 94°C for 10 seconds, annealing at 57–60°C for 30 seconds, and extension at 72°C for 1 minute (or 5 minutes for long PCR), followed by a final extension at 72°C for 10 minutes. Specific amplicons were separated and visualized on agarose gels. The PCR products were purified by isopropanol precipitation and/or using ExoSAP-IT (Affymetrix Inc., CA, USA). Using the PCR primers and internal primers, the purified PCR products were directly sequenced for complete coverage in both strand orientations with a BigDye Terminator v3.1 Cycle Sequencing Kit and a 3130 Genetic Analyzer (Applied Biosystems, CA, USA). Chromatograms were imported into FinchTV (Geospiza Inc., WA, USA) and analyzed.

Using PHASE v2.1 [49], [50], haplotypes were reconstructed from diploid sequence sets from which length-variant heterozygotes were excluded. The reconstructed haplotypes whose sites were inferred to have probabilities of less than 0.95 were not adopted. Haplotypes of the remaining unphased sequences were determined by sub-cloning the amplicons with a TOPO TA Cloning Kit (Invitrogen Corporation, CA, USA). In the 28 c*TAS2R*s, numbering of the nucleotide positions was assigned with the A of the ATG translation-start codon being taken as the first nucleotide. The sequences have been deposited with GenBank under accession numbers AB713189–AB713400.

### Identification of Variations in c*TAS2R*s

The human genome sequences (GRCh35/hg17 and GRCh37/hg19) and the chimpanzee genome sequences (CGSC 2.1.3/panTro3) were retrieved from the University of California, Santa Cruz Web site (http://genome.ucsc.edu/) as the reference for the whole-genome assemblies [51], [52]. By visual inspection, each c*TAS2R* sequence set was aligned with a corresponding h*TAS2R* haplotype annotated from GRCh35/hg17 and GRCh37/hg19. The variations were classified into synonymous change, non-synonymous change, loss of start codon, gain of stop codon, loss of stop codon, and indel (insertion and deletion). Median-joining networks of evolutionary relationships among the haplotypes were constructed using NETWORK v4.6 [53]. To identify ectopic gene conversion variations among c*TAS2R*s and the related pseudogenes, we aligned the sequences of all the inferred haplotypes and c*TAS2R* pseudogenes annotated from CGSC 2.1.3/panTro3 using E-INS-i in MAFFT v6.857b [54], and determined the length of gene conversion tracts between any 2 loci of the c*TAS2R*s and pseudogenes using the method reported by Betrán et al. [55] in DnaSP v5.1 [56].

### Intrasubspecific Diversity and Intersubspecific Divergence of c*TAS2R*s

We focused on the sequence set of the 46 western and the 10 eastern chimpanzees for analyses of intrasubspecific diversity and intersubspecific divergence. Several summary statistics were calculated for each c*TAS2R* using DnaSP v5.1 as follows. To assess the intersubspecific divergence level of each SNV, *F*_ST_ at each variable nucleotide site was calculated [57]. After eliminating all sites containing gaps, Nei and Li’s mean pairwise nucleotide differences per site within each subspecies (nucleotide diversity, *π*) and between subspecies (nucleotide divergence, *d*_XY_) were calculated [58]. Tajima’s *D* was also calculated in order to summarize haplotype frequency within each subspecies [59]. After eliminating all sites containing gaps and start and stop codons, mean pairwise nucleotide differences within each subspecies per synonymous site (synonymous diversity, *π*_S_) and per non-synonymous site (non-synonymous diversity, *π*_N_) were calculated by the method reported by Nei and Gojobori in order to measure the selective constraints on amino acid sequences [60]. Other statistical calculations were performed using R v2.14.0 (http://www.r-project.org/).

## Supporting Information

Figure S1 **A schematic representation of the junction sequence of the large-deletion variant.** The junction sequence is aligned with the corresponding sequence in CGSC 2.1.3/panTro3. The large deletion resulted in the whole-gene deletions of c*TAS2R43*, c*TAS2R46*, c*TAS2R63P*, and c*TAS2R64*. Vertical bars (|) within the alignment indicate identical nucleotides. Asterisks (*) within the alignment indicate different nucleotides. This variant sequence was isolated from an eastern chimpanzee (subject 156) using intC_F and int31-63_R as PCR and sequence primers with cutoff base calls of *Q*20. The sequence had no variable positions. We performed BLAT search against CGSC 2.1.3/panTro3 using the sequencing results as queries. The BLAT hits showed that the reverse sequence (623 bp) is consistent with positions of 11,256,872 to 11,324,827 on chromosome 12 (chr12) with identity 99.9% and spanning 67,956 bp with a large deletion from 11,257,350 to 11,324,679 (67,330 bp). The other BLAT hits did not show close alignments (<92% identity). The sequence around the large deletion contains retrotransposon sequences (*AluJr*, *L1MEg*, and *L1ME3B*).
(PDF)

Figure S2 **Monte Carlo simulations to reconstruct the haplotype distribution.** A histogram shows simulated distribution of a total expected number of shared haplotypes between western and eastern chimpanzees in the 28 c*TAS2R*s, assuming no differentiation of the 2 populations, given the sampling number of chromosomes (92 and 20, respectively) and the observed haplotypes with mean frequencies for the total metapopulation in each c*TAS2R* under 10,000 replicates. The probability that shared haplotypes were sampled in the observed number or less was estimated based on a fitted Gaussian distribution shown as a line graph. (A) Nucleotide haplotypes. (B) Protein haplotypes.
(PDF)

Table S1 Genotypes of subjects in this study.
(PDF)

Table S2 Haplotype frequency of each subspecies. Frequencies of non-functional haplotypes (pseudogenes and whole-gene deletions) are underlined.
(PDF)

Table S3 Genomic DNA samples in this study.
(PDF)

Table S4 PCR primers used in this study. These primers were designed based on CGSC 2.1.3/panTro3.
(PDF)
