# Supplementary material for: Eco-Geographical Diversification of Bitter Taste Receptor Genes (TAS2Rs) among Subspecies of Chimpanzees (Pan troglodytes)
Source: PLoS One. 2012 Aug 16;7(8):e43277. doi: 10.1371/journal.pone.0043277 (PMC3420883; doi:10.1371/journal.pone.0043277)
Supplement: Figure S1 — A schematic representation of the junction sequence of the large-deletion variant. The junction sequence is aligned with the corresponding sequence in CGSC 2.1.3/panTro3. The large deletion resulted in the whole-gene deletions of cTAS2R43, cTAS2R46, cTAS2R63P, and cTAS2R64. Vertical bars (|) within the alignment indicate identical nucleotides. Asterisks (*) within the alignment indicate different nucleotides. This variant sequence was isolated from an eastern chimpanzee (subject 156) using intC_F and int31-63_R as PCR and sequence primers with cutoff base calls of Q20. The sequence had no variable positions. We performed BLAT search against CGSC 2.1.3/panTro3 using the sequencing results as queries. The BLAT hits showed that the reverse sequence (623 bp) is consistent with positions of 11,256,872 to 11,324,827 on chromosome 12 (chr12) with identity 99.9% and spanning 67,956 bp with a large deletion from 11,257,350 to 11,324,679 (67,330 bp). The other BLAT hits did not show close alignments (<92% identity). The sequence around the large deletion contains retrotransposon sequences (AluJr, L1MEg, and L1ME3B). (PDF) [file pone.0043277.s001.pdf]

```
                          <-----Primer_int2_F----->
Chr12:11328763 ACAGATGTGA GAGGAATTAT CAAGAGGAAA AATACATTTA GTATAATCTC ATTTACATAA AGTTTGAAAA ACATTTTTAA TAATTGTAAT TTATTACATT

       Variant NNNNNNNNNN NNNNNNNNNN NNNNNNNNNN NNNNNNNNNN NNNNNNNNNN NNNNNNNNNN NNNNNNNNNN NNNNNNNNNN NNNNNNNNNN NNNNNNNNNN


Chr12:11328663 GGTGATAAAA CTCTACAGTG AAACATGAGT GATCATGATA ACAATTGGAA TAGTCAAGAG GGAGGAAAAT ATCAACAAGA AGACATATAT TGGGATTATC
                                 |||| |||||||||| |||||||||| |||||||||| |||||||||| |||||||||| |||||||||| ||||||||||
       Variant NNNNNNNNNN NNNNNNNNNN NNNNNNGAGT GATCATGATA ACAATTGGAA TAGTCAAGAG GGAGGAAAAT ATCAACAAGA AGACATATAT TGGGATTATC


Chr12:11328563 TGGATGGCTG GCAAATTCTT TTTTCTTACC AATGTGTGGT AACCTCCATG TTAATGTTAT AATTTTTTCT CTTTCTGTTT CTTTTGTGCT ATTACTCTCT
               |||||||||| |||||||||| |||||||||| |||||||||| |||||||||| |||||||||| |||||||||| |||||||||| |||||||||| ||||||||||
       Variant TGGATGGCTG GCAAATTCTT TTTTCTTACC AATGTGTGGT AACCTCCATG TTAATGTTAT AATTTTTTCT CTTTCTGTTT CTTTTGTGCT ATTACTCTCT


Chr12:11328463 ATATGTATGT TATGCTCCAC AATAAAAAAT GTTTAAAAAG CAGAAACATA AAGTATATTT CTAAGTATAA AATTAGGGTG ATACAGCATA TTCTCATAGA
               |||||||||| |||||||||| |||||||||| |||||||||| |||||||||| |||||||||| |||||||||| |||||||||| |||||||||| ||||||||||
       Variant ATATGTATGT TATGCTCCAC AATAAAAAAT GTTTAAAAAG CAGAAACATA AAGTATATTT CTAAGTATAA AATTAGGGTG ATACAGCATA TTCTCATAGA


Chr12:11328363 TACCATTGTA ATTAGAGTTT TTGCTGAATA AAGGAGAATA GAGCATTATG GTACTAAGAA ATCCAAATTA AAATTTAAAA CTTTTCTTTT TTTATTTTGA
               |||||||||| |||||||||| |||||||||| |||||||||| |||||||||| |||||||||| |||||||||| |||||||||| |||||||||| ||||||||||
       Variant TACCATTGTA ATTAGAGTTT TTGCTGAATA AAGGAGAATA GAGCATTATG GTACTAAGAA ATCCAAATTA AAATTTAAAA CTTTTCTTTT TTTATTTTGA


Chr12:11328263 GACAGAGTCT CACTCTGTTG CCCAGGCTGG AGTGCAGAAG CACAATCTTG GCTCAGTGCA GCCTCCGTCA CCTGGGCTCA AGTGATTCTC CCATCTCAGC
               |||||||||| |||||||||| |||||||||| |||||||||| |||||||||| |||||||||| |||||||||| |||||||||| |||||||||| ||||||||||
       Variant GACAGAGTCT CACTCTGTTG CCCAGGCTGG AGTGCAGAAG CACAATCTTG GCTCAGTGCA GCCTCCGTCA CCTGGGCTCA AGTGATTCTC CCATCTCAGC


Chr12:11328163 CTCCTGAATA TCTAGGACAA CAGGCGTGCA CCACCACGCC CTGCTAATTT TTTCCATTTT TTTTTTTTAG AGTCAGGGTT TTGCCATGTT GCCCAGGCTG
               |||||||||| |||||||||| |||||||||| |||||||||| |||||||||| |||||||||| ||||||
       Variant CTCCTGAATA TCTAGGACAA CAGGCGTGCA CCACCACGCC CTGCTAATTT TTTCCATTTT TTTTTTNNNN NNNNNNNNNN NNNNNNNNNN NNNNNNNNNN
```

Figure S1. A schematic representation of the junction sequence of the large-deletion variant. The junction sequence is aligned with the corresponding sequence in CGSC 2.1.3/panTro3. The large deletion resulted in the whole-gene deletions of c*TAS2R43*, c*TAS2R46*, c*TAS2R63P*, and c*TAS2R64*. Vertical bars (|) within the alignment indicate identical nucleotides. Asterisks (*) within the alignment indicate different nucleotides. This variant sequence was isolated from an eastern chimpanzee (subject 156) using intC_F and int31-63_R as PCR and sequence primers with cutoff base calls of *Q*20. The sequence had no variable positions. We performed BLAT search against CGSC 2.1.3/panTro3 using the sequencing results as queries. The BLAT hits showed that the reverse sequence (623 bp) is consistent with positions of 11,256,872 to 11,324,827 on chromosome 12 (chr12) with identity 99.9 % and spanning 67,956 bp with a large deletion from 11,257,350 to 11,324,679 (67,330 bp). The other BLAT hits did not show close alignments (<92 % identity). The sequence around the large deletion contains retrotransposon sequences (*AluJr*, *L1MEg*, and *L1ME3B*).

---//---

```
                          <----------------------------------------------AluJr----------------------------------------------
Chr12:11325112 AGAGTTGGAT TCTCACTCTG TTGTCCAGGC TGGAGTTCAG TGGTAGGATC ATGGCTTACT GCAGTCTGGA ACTCCTGGGC TCAAACCATC CTCCCACCTC

               ----------------------------------------------------AluJr----------------------------------------------------
Chr12:11325012 AGCCTTGTGA GTAGCTTCAC TATGCCTGAC TAACTTTTTC ATTCATTGTA GAGGCAGTTT CGCTGTGTTG CACAGGCTGG TCTTGAACTC CTGGGTGCTC

               ---------------------------AluJr--------------------------->                                <-----L1MEg------
Chr12:11324912 CTGCCCCAGC CTTACAAAAT GTTGGGGTTA TGGGTGTGAG CTACCCTGCC TGGCCAATAA AGGGTCTTAT TGAAAATATT AAGTTGAATA TGCAAGAAGG
                                                                                                    ||||| ||||||||||
       Variant NNNNNNNNNN NNNNNNNNNN NNNNNNNNNN NNNNNNNNNN NNNNNNNNNN NNNNNNNNNN NNNNNNNNNN NNNNNNNNNN NNNNNGAATA TGCAAGAAGG
               ----------------------------------------------------L1MEg----------------------------------------------------
Chr12:11324812 AATACAAATT AATGAAAGTG GTAAATATGT GCATAATTCT AAATGAATGT TTAGAAAATA ATAATGTCTT GTTGGGGTAA TTATACAATT GATAAGACAT
               |||||||||| |||||||||| |||||||||| |||||||||| |||||||||| ||||||***| |||||||||| |||||||||| |||||||||| ||||||||||
       Variant AATACAAATT AATGAAAGTG GTAAATATGT GCATAATTCT AAATGAATGT TTAGAA---A ATAATGTCTT GTTGGGGTAA TTATACAATT GATAAGACAT

               ---------------L1MEg--------------->
Chr12:11324712 ATGCAAATGG AAAAAAATAG AATGGAGGTA AAGGTGACAG GAGTAAGTTT AGTTAAATTA TTTTCTAAAC TTTTCTATGT CTATGAGGAG ATTTTGAACA
               |||||||||| |||||||||| |||||||||| |||******* ********** ********** ********** ********** ********** **********
       Variant ATGCAAATGG AAAAAAATAG AATGGAGGTA AAG------- ---------- ---------- ---------- ---------- ---------- ----------
                                                   <-----------------------------Large deletion-----------------------------

                          <---------------------------------------------L1ME3B----------------------------------------------
Chr12:11324612 ATGATAATAT AATTCTACTC ATAGGTATAT ATGCAAAGGA ATTGTATCAA ATGATATATG AAAGAATGTT CTAGTAGAAT TATTCATAAC TGTTCAAAAA
               ********** ********** ********** ********** ********** ********** ********** ********** ********** **********
       Variant ---------- ---------- ---------- ---------- ---------- ---------- ---------- ---------- ---------- ----------
               -----------------------------------------------Large deletion------------------------------------------------

               ---------------------------------------------------L1ME3B----------------------------------------------------
Chr12:11324512 AGAAACTGGC CAAATACATA TTAAGGTTGG ATGAATCATT ACAGTAATTC CATGCAATGG AACAATATAG AGAAGTGAAA AAAAATCACA TGTGCTTGCA
               ********** ********** ********** ********** ********** ********** ********** ********** ********** **********
       Variant ---------- ---------- ---------- ---------- ---------- ---------- ---------- ---------- ---------- ----------
               -----------------------------------------------Large deletion------------------------------------------------
```

---//---

Figure S1. Continued.

```
                 ---------------------------------------------------L1MEg---------------------------------------------------
Chr12:11257453 ATAATTCTAA ATGAATGTTT ACTATAAAAA ATAATAATAT CTTGTTGGGG TAATTATAAT TGATAAGACA TATGCAAATG GCAAAAAATA GAATGGAGGT
               ********** ********** ********** ********** ********** ********** ********** ********** ********** **********
       Variant ---------- ---------- ---------- ---------- ---------- ---------- ---------- ---------- ---------- ----------
               ----------------------------------------------Large deletion----------------------------------------------

               -->                                                                                      <--------L1ME3B--------
Chr12:11257353 AAAGGCGACA GGAGTAAGTT TAGTTAAATT ATTTCTTGAA CTTTTCTATG TCTATGAGGA GATTTTGAAC AATGTTAATG TAATTCCACT CATAGGTATA
               ****|||||| |||||||||| |||||||||| |||||||||| |||||||||| |||||||||| |||||||||| |||||||||| |||||||||| ||||||||||
       Variant ----GCGACA GGAGTAAGTT TAGTTAAATT ATTTCTTGAA CTTTTCTATG TCTATGAGGA GATTTTGAAC AATGTTAATG TAATTCCACT CATAGGTATA
               --->

               ---------------------------------------------------L1ME3B---------------------------------------------------
Chr12:11257253 TATGCCAAGG AATTGTATCA AATGATATAT GAAAGAATGT TCTAGTAGAA TTATTCATAA CTGTTCAAAA AAGAAGCTGG CCAAATACAT ATTAAGGTTG
               |||||||||| |||||||||| |||||||||| |||||||||| |||||||||| |||||||||| |||||||||| |||||||||| |||||||||| ||||||||||
       Variant TATGCCAAGG AATTGTATCA AATGATATAT GAAAGAATGT TCTAGTAGAA TTATTCATAA CTGTTCAAAA AAGAAGCTGG CCAAATACAT ATTAAGGTTG

               ---------------------------------------------------L1ME3B---------------------------------------------------
Chr12:11257153 GATGAATCAT TACAGTAATT CCGTGCAATG GAACATTATA GAAAAGTGAA AAAAATCACA TGTGCTTGCA ACTATGTGAC TAAATTTCAG AAGCATAATG
               |||||||||| |||||||||| |||||||||| |||||||||| |||||||||| |||||||||| |||*|||||| |||||||||| |||||||||| ||||||||||
       Variant GATGAATCAT TACAGTAATT CCGTGCAATG GAACATTATA GAAAAGTGAA AAAAATCACA TGTACTTGCA ACTATGTGAC TAAATTTCAG AAGCATAATG

               ---------------------------------------------------L1ME3B---------------------------------------------------
Chr12:11257053 TTGGGTCCAA GAAGCCACAA ACAAGAAGAA CATGTAGGAT TGCACCTACA CACAGTTCAA AGCAGGCCAG ACCAAGCTAC GGAGTTTAGG GTTGCATACC
               |||||||||| |||||||||| |||||||||| |||||||||| |||||||||| |||||||||| |||||||||| |||||||||| |||||||||| ||||||||||
       Variant TTGGGTCCAA GAAGCCACAA ACAAGAAGAA CATGTAGGAT TGCACCTACA CACAGTTCAA AGCAGGCCAG ACCAAGCTAC GGAGTTTAGG GTTGCATACC

               ---------------------------------------------------L1ME3B---------------------------------------------------
Chr12:11256953 TAGTTGGTAA AGTATAAAGA AAATTGAGGA AATAATCATC ATAAATTATG GATATTGCTT ATATCAGGAG ATGGGAAGAA GGGTTTAGGG GCTTGTAAGG
               |||||||||| |||||||||| |||||||||| |||||||||| |||||||||| |||||||||| |||||||||| |||||||||| ||
       Variant TAGTTGGTAA AGTATAAAGA AAATTGAGGA AATAATCATC ATAAATTATG GATATTGCTT ATATCAGGAG ATGGGAAGAA GGNNNNNNNN NNNNNNNNNN

               -----------------------------------L1ME3B------------------------------------>
Chr12:11256853 TTGCATGGAG GTGGACCTAG GCTGTTTTCG GTGTTCCTTG TCTTGCCCTG CATCATGGTT ACTTGAGTGT TTATGATACA CTGTGGTATT TCCCATTTTT

       Variant NNNNNNNNNN NNNNNNNNNN NNNNNNNNNN NNNNNNNNNN NNNNNNNNNN NNNNNNNNNN NNNNNNNNNN NNNNNNNNNN NNNNNNNNNN NNNNNNNNNN

                                                                          <-------Primer_44-63_R------->
Chr12:11256753 GTATTGGTTC ATTTCTAAGT GTGCATTATA ACTTTAATAT ACAAATTTGT AATTAGGAGA AATATCTCAT AGAAATTATT ATTACTCGTC TCATTATATA

       Variant NNNNNNNNNN NNNNNNNNNN NNNNNNNNNN NNNNNNNNNN NNNNNNNNNN NNNNNNNNNN NNNNNNNNNN NNNNNNNNNN NNNNNNNNNN NNNNNNNNNN
```

Figure S1. Continued.
