# Supplementary material for: Eco-Geographical Diversification of Bitter Taste Receptor Genes (TAS2Rs) among Subspecies of Chimpanzees (Pan troglodytes)
Source: PLoS One. 2012 Aug 16;7(8):e43277. doi: 10.1371/journal.pone.0043277 (PMC3420883; doi:10.1371/journal.pone.0043277)
Supplement: Figure S2 — Monte Carlo simulations to reconstruct the haplotype distribution. A histogram shows simulated distribution of a total expected number of shared haplotypes between western and eastern chimpanzees in the 28 cTAS2Rs, assuming no differentiation of the 2 populations, given the sampling number of chromosomes (92 and 20, respectively) and the observed haplotypes with mean frequencies for the total metapopulation in each cTAS2R under 10,000 replicates. The probability that shared haplotypes were sampled in the observed number or less was estimated based on a fitted Gaussian distribution shown as a line graph. (A) Nucleotide haplotypes. (B) Protein haplotypes. (PDF) [file pone.0043277.s002.pdf]

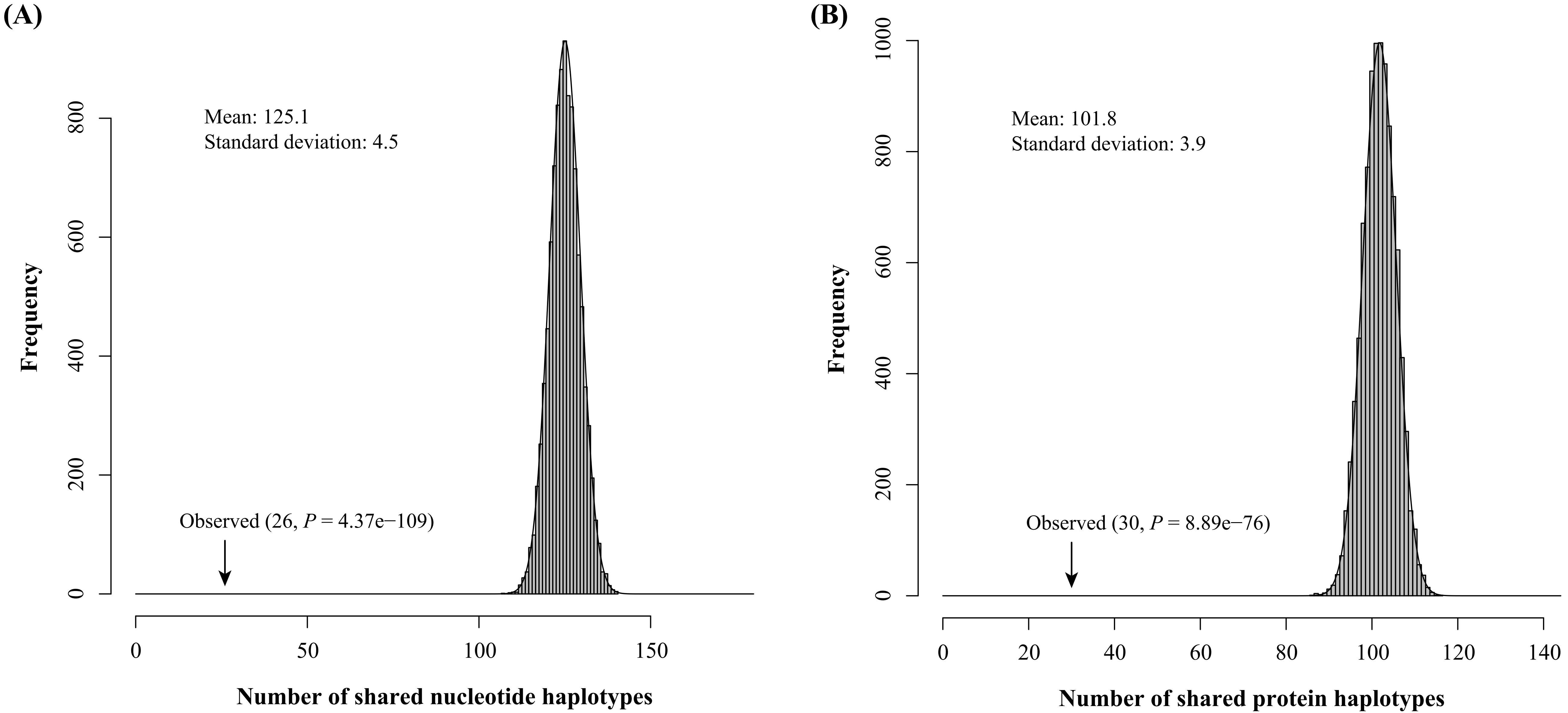

Figure S2. Monte Carlo simulations to reconstruct the haplotype distribution. A histogram shows simulated distribution of a total expected number of shared haplotypes between western and eastern chimpanzees in the 28 c*TAS2R*s, assuming no differentiation of the 2 populations, given the sampling number of chromosomes (92 and 20, respectively) and the observed haplotypes with mean frequencies for the total metapopulation in each c*TAS2R* under 10,000 replicates. The probability that shared haplotypes were sampled in the observed number or less was estimated based on a fitted Gaussian distribution shown as a line graph. (A) Nucleotide haplotypes. (B) Protein haplotypes.
