# Supplementary material for: Eco-Geographical Diversification of Bitter Taste Receptor Genes (TAS2Rs) among Subspecies of Chimpanzees (Pan troglodytes)
Source: PLoS One. 2012 Aug 16;7(8):e43277. doi: 10.1371/journal.pone.0043277 (PMC3420883; doi:10.1371/journal.pone.0043277)
Supplement: Table S1 — Genotypes of subjects in this study. (PDF) [file pone.0043277.s003.pdf]

Table S1. Genotypes of subjects in this study.

Local ID	Subspecies	Genotype of c*TAS2R*																											
		1	***2***	***3***	***4***	***5***	***7***	***8***	***9***	***10***	***13***	***14***	***16***	***19***	***20***	***30***	***31***	***38***	***39***	***40***	***41***	***42***	***43***	***45***	***46***	***50***	***60***	***62***	***64***
436	Western	1,1	1,1	1,2	1,2	1,1	1,1	1,2	1,2	1,1	1,2	1,1	1,1	1,1	1,2	1,2	1,2	1,1	1,2	1,1	1,1	1,1	1,2	1,1	1,2	1,2	1,2	1,2	1,1
437	Western	1,1	2,3	1,2	1,2	1,1	1,2	1,2	1,2	1,1	1,2	1,1	1,1	1,1	1,2	2,3	1,1	1,1	1,2	1,1	1,1	1,1	1,1	1,1	1,1	1,2	1,3	2,3	1,1
435	Western	1,2	1,2	2,3	2,2	1,1	1,1	1,1	1,1	1,1	1,3	1,2	1,1	1,1	1,3	3,3	1,2	1,2	1,1	1,1	1,1	1,2	2,3	1,2	2,3	1,3	1,1	1,1	1,2
434	Western	1,2	2,3	1,1	1,1	1,2	1,1	1,2	1,2	1,1	1,1	1,3	1,1	1,1	3,4	1,3	1,3	1,1	1,2	1,1	1,1	1,2	1,3	2,3	1,3	1,3	2,3	1,1	1,2
274	Western	1,2	2,3	1,2	1,2	1,1	1,1	1,2	1,2	1,1	4,4	1,1	1,1	1,2	2,2	1,3	1,4	1,1	1,1	1,1	1,1	1,1	1,1	1,4	1,1	2,2	1,1	2,2	1,1
432	Western	1,1	2,3	3,3	2,2	1,1	1,1	1,1	1,1	1,1	1,3	1,2	1,2	1,1	1,3	3,3	1,2	2,2	1,1	1,1	1,1	1,2	2,3	1,2	1,3	1,3	3,4	1,1	1,2
193	Western	1,2	1,1	1,1	1,1	1,1	1,1	1,2	1,2	1,1	1,1	1,2	1,2	1,1	1,4	3,3	1,2	1,3	1,1	1,1	1,1	1,2	2,4	1,5	1,1	1,1	1,3	1,1	1,3
110	Western	1,1	3,3	1,2	1,2	1,1	1,1	1,2	1,2	1,1	4,4	1,2	1,1	1,1	2,3	1,3	1,3	1,1	1,1	1,1	1,2	1,2	3,5	2,4	1,3	2,3	1,3	1,2	1,2
132	Western	1,1	2,2	2,3	2,2	1,2	1,2	1,1	1,1	1,1	1,1	2,2	1,2	1,2	1,2	3,3	2,4	1,2	1,1	1,1	1,1	1,3	2,3	1,2	1,4	1,2	1,2	1,2	1,2
119	Western	1,2	1,3	3,3	2,2	1,1	1,1	1,2	1,2	1,1	1,1	2,3	1,1	1,2	2,3	3,3	1,4	1,2	1,1	1,1	1,1	1,2	3,5	1,2	1,3	2,3	1,3	1,1	2,4
131	Western	1,1	2,2	3,3	2,2	1,1	1,1	1,2	1,2	1,1	1,5	1,2	1,2	1,1	4,4	1,3	1,1	1,2	1,2	1,1	1,1	1,1	1,4	1,5	1,1	1,1	1,3	1,2	1,3
143	Western	1,1	2,2	1,2	1,2	1,1	1,2	1,1	1,1	1,1	1,1	2,3	1,1	1,1	1,3	3,3	2,3	1,2	1,1	1,1	1,1	1,2	2,3	1,2	1,3	1,3	1,3	1,1	1,2
146	Western	1,1	2,3	1,1	1,1	1,1	1,1	2,3	1,2	1,1	1,1	1,2	1,2	1,1	1,4	3,3	1,2	1,1	1,1	1,1	1,1	1,1	2,4	1,5	1,1	1,1	2,3	1,1	1,3
449	Western	1,2	1,3	1,3	1,2	1,1	1,1	1,3	1,1	1,1	1,1	1,1	1,2	1,1	4,4	1,3	1,1	1,2	1,1	1,1	1,1	1,1	2,4	1,5	1,1	1,1	1,4	1,1	1,3
135	Western	2,2	1,2	1,3	1,2	1,2	1,1	2,3	1,2	1,1	1,2	1,1	1,1	1,1	1,4	1,3	1,2	1,2	1,1	1,1	1,1	1,4	1,2	1,1	1,1	1,1	3,3	1,1	1,1
448	Western	1,2	2,3	1,3	1,2	1,1	1,1	1,1	1,1	1,1	1,1	1,2	1,1	1,2	1,2	3,3	2,4	1,1	1,1	1,1	1,2	1,3	2,3	1,2	1,5	1,2	3,3	1,1	1,2
268	Western	1,2	2,2	1,3	1,2	1,1	1,1	2,2	2,2	1,1	1,1	2,2	1,1	1,1	4,4	3,3	1,1	1,2	1,1	1,1	1,2	1,1	4,4	5,5	1,1	1,1	1,1	1,1	3,3
451	Western	1,1	1,2	1,1	1,1	1,1	1,1	1,1	1,1	1,2	1,2	1,1	1,1	1,1	1,2	1,3	1,2	1,4	1,1	1,1	1,1	1,1	1,2	1,1	1,1	1,2	1,1	1,2	1,1
445	Western	1,1	1,2	1,3	1,2	1,2	1,1	1,1	1,1	1,1	1,1	1,1	1,2	1,2	2,2	3,3	1,4	1,1	1,1	1,1	1,1	1,3	2,3	1,2	1,4	1,2	1,1	1,2	1,2
159	Western	1,1	1,3	3,3	2,2	1,1	1,2	1,1	1,1	1,1	1,3	2,2	1,1	1,1	1,1	3,3	2,2	1,1	1,1	1,1	1,1	1,1	2,2	1,1	1,2	1,1	1,3	1,2	1,1
170	Western	1,1	1,2	2,3	2,2	1,1	1,1	1,1	1,1	1,1	1,6	4,4	1,1	1,1	1,4	1,3	1,2	1,1	1,2	1,1	1,1	1,4	1,2	1,1	1,1	1,1	1,3	1,2	1,1
454	Western	1,2	2,2	1,1	1,1	1,1	1,1	1,1	1,1	1,1	1,3	4,4	1,1	1,1	1,4	3,3	2,2	1,1	1,1	1,1	1,1	1,1	1,2	1,1	1,1	1,4	3,3	1,1	1,4
269	Western	1,2	3,3	1,3	1,2	1,1	1,1	1,2	1,2	1,1	2,3	1,1	1,2	1,2	2,4	1,3	1,4	1,2	1,1	1,1	1,1	1,3	1,3	1,2	1,4	1,2	3,3	1,1	1,2
457	Western	1,2	1,2	1,3	1,2	1,1	1,2	1,1	1,1	1,1	1,1	1,1	1,1	1,1	1,3	3,3	2,3	1,2	2,2	1,1	1,1	1,2	2,3	1,2	1,3	1,3	1,2	1,2	1,2
458	Western	1,1	2,3	1,1	1,1	1,1	1,1	1,1	1,1	1,1	1,1	1,3	1,1	1,1	3,3	3,3	1,3	1,1	1,1	1,1	1,2	2,2	3,3	2,2	3,3	3,3	1,3	1,2	2,2
204	Western	1,1	2,3	1,3	1,2	1,1	1,1	1,1	1,1	1,1	1,3	2,2	1,1	1,2	2,3	3,3	3,4	1,3	1,2	1,1	1,1	2,3	3,3	2,2	3,4	2,3	1,1	1,1	2,2
205	Western	1,1	2,3	1,2	1,2	1,1	1,1	1,2	1,2	1,1	1,1	2,2	1,1	1,1	2,4	3,3	1,1	1,1	1,1	1,1	1,1	1,1	2,4	1,5	1,1	1,1	1,3	2,2	1,3
206	Western	1,3	1,3	1,3	1,2	1,1	1,1	1,1	1,1	1,1	1,4	1,1	1,1	1,1	2,4	1,3	1,2	1,1	1,1	1,1	1,1	1,1	1,3	2,4	1,5	1,2	1,3	1,2	1,2
211	Western	1,1	1,2	1,2	1,2	1,1	1,1	1,2	1,2	1,1	1,3	2,2	1,1	2,2	2,2	3,3	4,4	1,3	1,1	1,1	1,2	1,3	1,3	1,2	1,4	2,2	1,1	1,2	2,4
212	Western	1,2	2,3	1,2	1,2	1,1	1,2	1,2	1,1	1,1	1,1	1,2	1,1	1,1	1,4	1,3	1,2	1,3	1,1	1,1	1,1	1,1	1,2	1,1	1,1	1,1	1,3	1,1	1,1
459	Western	1,2	1,2	3,4	1,2	1,1	1,1	1,2	2,2	1,1	1,2	1,1	1,2	1,1	2,2	2,3	1,1	1,4	1,1	1,1	1,1	1,1	1,2	1,1	1,1	2,2	1,2	1,2	1,1

Table S1. Continued.

Local ID	Subspecies	Genotype of c*TAS2R*																											
		1	***2***	***3***	***4***	***5***	***7***	***8***	***9***	***10***	***13***	***14***	***16***	***19***	***20***	***30***	***31***	***38***	***39***	***40***	***41***	***42***	***43***	***45***	***46***	***50***	***60***	***62***	***64***
461	Western	1,2	1,2	2,3	2,2	1,1	1,2	1,1	1,1	1,1	2,3	1,2	1,2	1,1	1,4	1,3	1,2	1,1	1,1	1,1	1,1	1,4	2,2	1,1	1,1	1,1	3,3	1,1	1,1
456	Western	1,2	1,3	3,3	2,2	1,1	1,1	1,1	1,1	1,1	1,3	1,1	1,1	1,1	1,1	3,3	2,2	1,3	1,1	1,1	1,1	1,1	1,2	1,1	1,1	1,1	2,3	1,1	1,1
455	Western	1,1	2,3	1,2	1,2	1,1	1,1	1,1	1,1	1,1	1,1	1,1	1,2	1,1	4,4	3,3	2,2	1,2	1,1	1,1	1,1	1,1	3,3	2,2	1,1	1,1	3,3	1,1	2,2
303	Western	1,2	1,1	1,3	1,2	1,1	1,2	1,1	1,1	1,1	1,1	2,3	1,1	1,1	1,3	1,3	1,3	1,2	1,2	1,1	1,1	1,2	1,3	1,2	1,3	1,3	1,3	1,2	1,2
307	Western	1,1	3,3	1,3	1,2	1,1	1,2	1,2	1,2	1,1	1,1	2,2	1,1	1,1	4,4	3,4	1,1	1,1	1,1	1,1	1,1	1,1	4,4	5,5	1,1	1,1	2,4	1,1	3,3
276	Western	1,1	2,3	1,3	1,2	1,1	1,1	1,2	1,2	1,1	1,1	1,3	1,1	1,1	3,3	3,3	3,4	1,4	1,1	1,1	1,1	2,3	3,3	2,2	3,3	2,3	1,2	1,1	1,2
277	Western	1,2	3,3	1,1	1,1	1,2	1,1	1,4	1,1	1,1	1,1	1,2	1,1	1,1	1,4	3,3	2,2	1,1	1,1	1,1	1,1	1,1	2,2	1,1	1,2	1,1	1,1	2,4	1,1
278	Western	1,1	1,3	1,1	1,1	1,1	1,2	1,2	1,2	1,1	1,1	2,3	1,1	1,1	1,3	3,3	2,3	1,1	1,2	1,1	1,1	1,2	2,3	1,2	1,3	1,3	1,3	2,4	1,2
279	Western	1,1	1,3	2,2	2,2	1,1	1,1	1,2	1,2	1,1	2,3	1,1	1,2	1,1	1,2	1,3	1,2	2,2	1,1	1,1	1,1	2,2	1,2	1,1	1,2	1,2	1,3	1,2	1,1
280	Western	1,1	1,3	1,1	1,1	2,2	1,1	1,2	1,2	1,1	1,3	1,2	1,2	1,1	1,4	3,3	2,2	1,2	1,2	1,1	1,1	1,1	2,3	1,2	1,5	1,1	1,2	1,2	1,2
281	Western	1,2	3,3	1,2	1,2	1,1	1,1	1,2	1,2	1,1	1,2	1,1	1,1	1,1	2,3	1,3	1,1	1,3	1,1	1,1	1,1	1,2	1,3	1,2	1,3	2,3	1,2	1,1	1,2
305	Western	1,2	3,3	1,3	1,2	1,1	1,2	1,1	1,1	1,1	1,1	1,1	1,1	1,1	1,4	3,3	2,2	2,2	1,1	1,1	1,1	1,1	2,3	1,2	1,5	1,1	3,4	1,1	1,2
283	Western	1,1	1,2	1,3	1,2	1,1	1,2	1,1	1,1	1,1	1,3	2,2	1,1	1,1	1,1	3,3	1,2	1,1	1,2	1,1	1,1	1,4	2,4	1,5	1,1	1,1	1,2	1,1	1,3
201	Western	1,1	3,3	1,3	1,2	1,1	1,1	1,2	1,2	1,1	1,1	1,1	1,2	1,2	2,2	1,3	1,4	1,1	1,2	1,1	1,1	1,1	1,3	1,2	1,4	2,2	1,3	1,2	1,2
358	Western	1,2	1,2	1,2	1,2	1,1	1,2	1,2	1,2	1,1	1,1	2,2	1,2	1,1	1,4	1,3	1,1	1,1	1,1	1,1	1,1	1,1	1,4	1,5	1,1	1,1	1,1	1,2	1,3
220	Eastern	4,4	2,2	5,5	3,3	1,1	3,4	1,1	1,1	1,1	1,1	1,1	3,4	3,4	5,5	3,3	1,5	2,2	1,3	1,2	3,3	1,5	6,7	1,6	1,6	5,6	5,5	5,5	1,2
153	Eastern	4,5	2,4	1,6	1,1	1,1	3,5	1,1	1,1	1,3	1,1	1,5	1,3	1,3	4,5	3,3	1,2	2,5	1,1	1,1	3,3	1,6	6,*Δ*	6,7	6,*Δ*	1,5	5,6	5,6	2,*Δ*
140	Eastern	4,6	3,3	1,5	1,3	1,1	4,6	1,5	1,3	1,1	1,1	6,6	1,1	5,6	4,6	3,5	6,7	2,2	1,1	1,2	3,4	1,1	8,9	1,1	1,7	5,5	5,5	5,7	2,5
249	Eastern	4,7	3,3	1,1	1,1	1,1	1,7	1,6	1,4	1,1	1,1	1,1	1,1	4,5	5,5	3,3	1,5	2,6	1,4	1,1	3,3	5,6	7,8	1,1	1,7	6,6	5,5	5,5	1,6
4	Eastern	7,8	3,3	1,5	1,4	1,1	3,6	6,6	1,5	1,1	1,1	1,1	1,3	3,5	5,5	3,3	1,1	2,2	1,4	1,1	3,3	1,6	6,8	1,6	6,7	5,6	5,5	5,8	2,6
182	Eastern	4,5	3,4	5,5	3,3	1,1	7,8	1,6	5,6	1,1	1,7	1,1	1,5	4,5	5,7	3,6	5,6	2,5	3,4	1,2	3,3	1,7	7,8	1,8	1,7	1,6	5,5	5,9	1,5
250	Eastern	7,9	3,3	1,1	1,1	1,1	1,6	1,5	1,3	1,1	1,1	1,1	1,3	5,6	4,5	3,5	1,7	2,2	1,1	1,3	3,3	1,6	8,9	1,1	1,7	5,6	5,5	5,8	6,7
156	Eastern	4,5	3,3	1,7	1,1	1,1	5,6	1,6	5,5	1,3	1,1	5,5	1,3	1,1	4,4	3,3	2,2	6,6	1,1	1,1	3,3	6,6	*Δ*,*Δ*	7,7	*Δ*,*Δ*	1,1	5,5	10,10	*Δ*,*Δ*
147	Eastern	4,9	3,3	1,1	1,1	1,1	1,7	1,6	1,5	1,1	1,1	1,1	1,1	5,6	4,5	3,5	1,7	2,2	1,4	1,1	3,3	1,7	8,9	1,1	1,7	5,6	5,7	5,6	6,7
282	Eastern	4,7	2,3	1,5	1,3	1,1	3,8	1,1	1,1	4,5	1,1	1,5	1,6	4,4	4,5	3,3	1,5	2,6	1,4	1,2	3,3	5,5	7,10	1,9	1,7	6,6	5,5	7,9	1,8
148	Central	5,6	3,5	1,1	1,5	1,3	8,9	1,7	1,7	1,1	1,1	1,1	1,4	1,4	4,6	3,3	1,8	2,2	1,5	1,1	1,1	1,1	1,10	8,10	1,7	1,7	8,9	5,9	1,8
11	Central	10,11	2,2	1,5	1,6	1,1	6,8	1,6	5,5	1,1	1,1	1,1	1,1	1,7	5,6	3,5	1,8	2,2	1,6	2,4	3,3	1,8	1,11	10,10	1,7	1,1	1,9	2,5	1,6
150[b]	NC	6,6	2,2	1,1	1,1	1,4	6,10	5,5	8,9	1,1	8,8	7,8	1,1	6,8	3,4	3,6	1,7	2,2	7,8	1,1	1,1	7,9	7,12	1,1	1,8	3,6	3,9	5,11	1,9

[a]The pair of numbers indicates combination of the 2 haplotypes, numbered in GenBank (AB713189–AB713400). *Δ* indicates the whole gene deletion.

[b]The individual was only maternally identified as a Nigerian-Cameroonian (NC) chimpanzee due to the lack of information about the antecedents in captivity.
