# Supplementary material for: Eco-Geographical Diversification of Bitter Taste Receptor Genes (TAS2Rs) among Subspecies of Chimpanzees (Pan troglodytes)
Source: PLoS One. 2012 Aug 16;7(8):e43277. doi: 10.1371/journal.pone.0043277 (PMC3420883; doi:10.1371/journal.pone.0043277)
Supplement: Table S2 — Haplotype frequency of each subspecies. Frequencies of non-functional haplotypes (pseudogenes and whole-gene deletions) are underlined. (PDF) [file pone.0043277.s004.pdf]

Table S2. Haplotype frequency of each subspecies. Frequencies of non-functional haplotypes (pseudogenes and whole-gene deletions) are underlined.

	Haplotype number												
Western (*N*=92)	1	2	3	4	5	6	7	8	9	10	11	12	*Δ*
c*TAS2R1*	69 (75 %)	22 (24 %)	1 (1 %)	0	0	0	0	0	0	0	0	–	–
c*TAS2R2*	25 (27 %)	33 (36 %)	34 (37 %)	0	0	–	–	–	–	–	–	–	–
c*TAS2R3*	44 (48 %)	17 (18 %)	30 (33 %)	1 (1 %)	0	0	0	–	–	–	–	–	–
c*TAS2R4*	45 (49 %)	47 (51 %)	0	0	0	0	–	–	–	–	–	–	–
c*TAS2R5*	85 (92 %)	7 (8 %)	0	0	–	–	–	–	–	–	–	–	–
c*TAS2R7*	79 (86 %)	13 (14 %)	0	0	0	0	0	0	0	0	–	–	–
c*TAS2R8*	63 (68 %)	25 (27 %)	3 (3 %)	1 (1 %)	0	0	0	–	–	–	–	–	–
c*TAS2R9*	67 (73 %)	25 (27 %)	0	0	0	0	0	0	0	–	–	–	–
c*TAS2R10*	91 (99 %)	1 (1 %)	0	0	0	–	–	–	–	–	–	–	–
c*TAS2R13*	64 (70 %)	9 (10 %)	12 (13 %)	5 (5 %)	1 (1 %)	1 (1 %)	0	0	–	–	–	–	–
c*TAS2R14*	48 (52 %)	33 (36 %)	7 (8 %)	4 (4 %)	0	0	0	0	–	–	–	–	–
c*TAS2R16*	77 (84 %)	15 (16 %)	0	0	0	0	–	–	–	–	–	–	–
c*TAS2R19*	82 (89 %)	10 (11 %)	0	0	0	0	0	0	–	–	–	–	–
c*TAS2R20*	29 (32 %)	23 (25 %)	15 (16 %)	25 (27 %)	0	0	0	–	–	–	–	–	–
c*TAS2R30*	18 (20 %)	3 (3 %)	70 (76 %)	1 (1 %)	0	0	–	–	–	–	–	–	–
c*TAS2R31*	40 (43 %)	32 (35 %)	9 (10 %)	11 (12 %)	0	0	0	0	–	–	–	–	–
c*TAS2R38*	64 (70 %)	19 (21 %)	6 (7 %)	3 (3 %)	0	0	–	–	–	–	–	–	–
c*TAS2R39*	79 (86 %)	13 (14 %)	0	0	0	0	0	0	–	–	–	–	–
c*TAS2R40*	92 (100 %)	0	0	0	–	–	–	–	–	–	–	–	–
c*TAS2R41*	87 (95 %)	5 (5 %)	0	0	–	–	–	–	–	–	–	–	–
c*TAS2R42*	64 (70 %)	17 (18 %)	7 (8 %)	4 (4 %)	0	0	0	0	0	–	–	–	–
c*TAS2R43*	22 (24 %)	30 (33 %)	27 (29 %)	11 (12 %)	2 (2 %)	0	0	0	0	0	0	0	0
c*TAS2R45*	50 (54 %)	27 (29 %)	1 (1 %)	3 (3 %)	11 (12 %)	0	0	0	0	0	–	–	–
c*TAS2R46*	62 (67 %)	5 (5 %)	15 (16 %)	6 (7 %)	4 (4 %)	0	0	0	–	–	–	–	0
c*TAS2R50*	55 (60 %)	22 (24 %)	14 (15 %)	1 (1 %)	0	0	0	–	–	–	–	–	–
c*TAS2R60*	43 (47 %)	12 (13 %)	33 (36 %)	4 (4 %)	0	0	0	0	0	–	–	–	–
c*TAS2R62*	64 (70 %)	25 (27 %)	1 (1 %)	2 (2 %)	0	0	0	0	0	0	0	–	–
c*TAS2R64*	52 (57 %)	26 (28 %)	11 (12 %)	3 (3 %)	0	0	0	0	0	–	–	–	0

Table S2. Continued.

Eastern (N=20)	Haplotype number											
	1	2	3	4	5	6	7	8	9	10	11	12	Δ
c*TAS2R1*	0	0	0	9 (45 %)	3 (15 %)	1 (5 %)	4 (20 %)	1 (5 %)	2 (10 %)	0	0	–	–
c*TAS2R2*	0	4 (20 %)	14 (70 %)	2 (10 %)	0	–	–	–	–	–	–	–	–
c*TAS2R3*	11 (55 %)	0	0	0	7 (35 %)	1 (5 %)	1 (5 %)	–	–	–	–	–	–
c*TAS2R4*	13 (65 %)	0	6 (30 %)	1 (5 %)	0	0	–	–	–	–	–	–	–
c*TAS2R5*	20 (100 %)	0	0	0	–	–	–	–	–	–	–	–	–
c*TAS2R7*	3 (15 %)	0	4 (20 %)	2 (10 %)	2 (10 %)	4 (20 %)	3 (15 %)	2 (10 %)	0	0	–	–	–
c*TAS2R8*	12 (60 %)	0	0	0	2 (10 %)	6 (30 %)	0	–	–	–	–	–	–
c*TAS2R9*	11 (55 %)	0	2 (10 %)	1 (5 %)	5 (25 %)	1 (5 %)	0	0	0	–	–	–	–
c*TAS2R10*	16 (80 %)	0	2 (10 %)	1 (5 %)	1 (5 %)	–	–	–	–	–	–	–	–
c*TAS2R13*	19 (95 %)	0	0	0	0	0	1 (5 %)	0	–	–	–	–	–
c*TAS2R14*	14 (70 %)	0	0	0	4 (20 %)	2 (10 %)	0	0	–	–	–	–	–
c*TAS2R16*	12 (60 %)	0	5 (25 %)	1 (5 %)	1 (5 %)	1 (5 %)	–	–	–	–	–	–	–
c*TAS2R19*	3 (15 %)	0	3 (15 %)	5 (25 %)	6 (30 %)	3 (15 %)	0	0	–	–	–	–	–
c*TAS2R20*	0	0	0	7 (35 %)	11 (55 %)	1 (5 %)	1 (5 %)	–	–	–	–	–	–
c*TAS2R30*	0	0	16 (80 %)	0	3 (15 %)	1 (5 %)	–	–	–	–	–	–	–
c*TAS2R31*	8 (40 %)	3 (15 %)	0	0	4 (20 %)	2 (10 %)	3 (15 %)	0	–	–	–	–	–
c*TAS2R38*	0	14 (70 %)	0	0	2 (10 %)	4 (20 %)	–	–	–	–	–	–	–
c*TAS2R39*	13 (65 %)	0	2 (10 %)	5 (25 %)	0	0	0	0	–	–	–	–	–
c*TAS2R40*	15 (75 %)	4 (20 %)	1 (5 %)	0	–	–	–	–	–	–	–	–	–
c*TAS2R41*	0	0	19 (95 %)	1 (5 %)	–	–	–	–	–	–	–	–	–
c*TAS2R42*	8 (40 %)	0	0	0	4 (20 %)	6 (30 %)	2 (10 %)	0	0	–	–	–	–
c*TAS2R43*	0	0	0	0	0	3 (15 %)	4 (20 %)	6 (30 %)	3 (15 %)	1 (5 %)	0	0	3 (15 %)
c*TAS2R45*	12 (60 %)	0	0	0	0	3 (15 %)	3 (15 %)	1 (5 %)	1 (5 %)	0	–	–	–
c*TAS2R46*	7 (35 %)	0	0	0	0	3 (15 %)	7 (35 %)	0	–	–	–	–	3 (15 %)
c*TAS2R50*	4 (20 %)	0	0	0	7 (35 %)	9 (45 %)	0	–	–	–	–	–	–
c*TAS2R60*	0	0	0	0	18 (90 %)	1 (5 %)	1 (5 %)	0	0	–	–	–	–
c*TAS2R62*	0	0	0	0	10 (50 %)	2 (10 %)	2 (10 %)	2 (10 %)	2 (10 %)	2 (10 %)	0	–	–
c*TAS2R64*	4 (20 %)	4 (20 %)	0	0	2 (10 %)	4 (20 %)	2 (10 %)	1 (5 %)	0	–	–	–	3 (15 %)

Table S2. Continued.

Central (*N*=4)	Haplotype number												
	1	2	3	4	5	6	7	8	9	10	11	12	*Δ*
c*TAS2R1*	0	0	0	0	1 (25 %)	1 (25 %)	0	0	0	1 (25 %)	1 (25 %)	–	–
c*TAS2R2*	0	2 (50 %)	1 (25 %)	0	1 (25 %)	–	–	–	–	–	–	–	–
c*TAS2R3*	3 (75 %)	0	0	0	1 (25 %)	0	0	–	–	–	–	–	–
c*TAS2R4*	2 (50 %)	0	0	0	1 (25 %)	1 (25 %)	–	–	–	–	–	–	–
c*TAS2R5*	3 (75 %)	0	1 (25 %)	0	–	–	–	–	–	–	–	–	–
c*TAS2R7*	0	0	0	0	0	1 (25 %)	0	2 (50 %)	1 (25 %)	0	–	–	–
c*TAS2R8*	2 (50 %)	0	0	0	0	1 (25 %)	1 (25 %)	–	–	–	–	–	–
c*TAS2R9*	1 (25 %)	0	0	0	2 (50 %)	0	1 (25 %)	0	0	–	–	–	–
c*TAS2R10*	4 (100 %)	0	0	0	0	–	–	–	–	–	–	–	–
c*TAS2R13*	4 (100 %)	0	0	0	0	0	0	0	–	–	–	–	–
c*TAS2R14*	4 (100 %)	0	0	0	0	0	0	0	–	–	–	–	–
c*TAS2R16*	3 (75 %)	0	0	1 (25 %)	0	0	–	–	–	–	–	–	–
c*TAS2R19*	2 (50 %)	0	0	1 (25 %)	0	0	1 (25 %)	0	–	–	–	–	–
c*TAS2R20*	0	0	0	1 (25 %)	1 (25 %)	2 (50 %)	0	–	–	–	–	–	–
c*TAS2R30*	0	0	3 (75 %)	0	1 (25 %)	0	–	–	–	–	–	–	–
c*TAS2R31*	2 (50 %)	0	0	0	0	0	0	2 (50 %)	–	–	–	–	–
c*TAS2R38*	0	4 (100 %)	0	0	0	0	–	–	–	–	–	–	–
c*TAS2R39*	2 (50 %)	0	0	0	1 (25 %)	1 (25 %)	0	0	–	–	–	–	–
c*TAS2R40*	2 (50 %)	1 (25 %)	0	1 (25 %)	–	–	–	–	–	–	–	–	–
c*TAS2R41*	2 (50 %)	0	2 (50 %)	0	–	–	–	–	–	–	–	–	–
c*TAS2R42*	3 (75 %)	0	0	0	0	0	0	1 (25 %)	0	–	–	–	–
c*TAS2R43*	2 (50 %)	0	0	0	0	0	0	0	0	1 (25 %)	1 (25 %)	0	0
c*TAS2R45*	0	0	0	0	0	0	0	1 (25 %)	0	3 (75 %)	–	–	–
c*TAS2R46*	2 (50 %)	0	0	0	0	0	2 (50 %)	0	–	–	–	–	0
c*TAS2R50*	3 (75 %)	0	0	0	0	0	1 (25 %)	–	–	–	–	–	–
c*TAS2R60*	1 (25 %)	0	0	0	0	0	0	1 (25 %)	2 (50 %)	–	–	–	–
c*TAS2R62*	0	1 (25 %)	0	0	2 (50 %)	0	0	0	1 (25 %)	0	0	–	–
c*TAS2R64*	2 (50 %)	0	0	0	0	1 (25 %)	0	1 (25 %)	0	–	–	–	0

Table S2. Continued.

NC[a] (*N*=2)	Haplotype number 1	2	3	4	5	6	7	8	9	10	11	12	*Δ*
c*TAS2R1*	0	0	0	0	0	2 (100 %)	0	0	0	0	0	–	–
c*TAS2R2*	0	2 (100 %)	0	0	0	–	–	–	–	–	–	–	–
c*TAS2R3*	2 (100 %)	0	0	0	0	0	0	–	–	–	–	–	–
c*TAS2R4*	2 (100 %)	0	0	0	0	0	–	–	–	–	–	–	–
c*TAS2R5*	1 (50 %)	0	0	1 (50 %)	–	–	–	–	–	–	–	–	–
c*TAS2R7*	0	0	0	0	0	1 (50 %)	0	0	0	1 (50 %)	–	–	–
c*TAS2R8*	0	0	0	0	2 (100 %)	0	0	–	–	–	–	–	–
c*TAS2R9*	0	0	0	0	0	0	0	1 (50 %)	1 (50 %)	–	–	–	–
c*TAS2R10*	2 (100 %)	0	0	0	0	–	–	–	–	–	–	–	–
c*TAS2R13*	0	0	0	0	0	0	0	2 (100 %)	–	–	–	–	–
c*TAS2R14*	0	0	0	0	0	0	1 (50 %)	1 (50 %)	–	–	–	–	–
c*TAS2R16*	2 (100 %)	0	0	0	0	0	–	–	–	–	–	–	–
c*TAS2R19*	0	0	0	0	0	1 (50 %)	0	1 (50 %)	–	–	–	–	–
c*TAS2R20*	0	0	1 (50 %)	1 (50 %)	0	0	0	–	–	–	–	–	–
c*TAS2R30*	0	0	1 (50 %)	0	0	1 (50 %)	–	–	–	–	–	–	–
c*TAS2R31*	1 (50 %)	0	0	0	0	0	1 (50 %)	0	–	–	–	–	–
c*TAS2R38*	0	2 (100 %)	0	0	0	0	–	–	–	–	–	–	–
c*TAS2R39*	0	0	0	0	0	0	1 (50 %)	1 (50 %)	–	–	–	–	–
c*TAS2R40*	2 (100 %)	0	0	0	–	–	–	–	–	–	–	–	–
c*TAS2R41*	2 (100 %)	0	0	0	–	–	–	–	–	–	–	–	–
c*TAS2R42*	0	0	0	0	0	0	1 (50 %)	0	1 (50 %)	–	–	–	–
c*TAS2R43*	0	0	0	0	0	0	1 (50 %)	0	0	0	0	1 (50 %)	0
c*TAS2R45*	2 (100 %)	0	0	0	0	0	0	0	0	0	–	–	–
c*TAS2R46*	1 (50 %)	0	0	0	0	0	0	1 (50 %)	–	–	–	–	0
c*TAS2R50*	0	0	1 (50 %)	0	0	1 (50 %)	0	–	–	–	–	–	–
c*TAS2R60*	0	0	1 (50 %)	0	0	0	0	0	1 (50 %)	–	–	–	–
c*TAS2R62*	0	0	0	0	1 (50 %)	0	0	0	0	0	1 (50 %)	–	–
c*TAS2R64*	1 (50 %)	0	0	0	0	0	0	0	1 (50 %)	–	–	–	0

[a]Subspecies of a Nigerian-Cameroonian (NC) chimpanzee was identified only maternally due to a lack of information about the antecedents in captivity.
