# Supplementary material for: Eco-Geographical Diversification of Bitter Taste Receptor Genes (TAS2Rs) among Subspecies of Chimpanzees (Pan troglodytes)
Source: PLoS One. 2012 Aug 16;7(8):e43277. doi: 10.1371/journal.pone.0043277 (PMC3420883; doi:10.1371/journal.pone.0043277)
Supplement: Table S3 — Genomic DNA samples in this study. (PDF) [file pone.0043277.s005.pdf]

Table S3. Genomic DNA samples in this study.

Local ID	Sex	Subspecies	DNA origin
220	Female	Eastern	Hair[a]
153	Female	Eastern	Hair[a]
140	Female	Eastern	Hair[a]
249	Female	Eastern	Hair[a]
4	Female	Eastern	Hair[a]
182	Female	Eastern	Hair[a]
250	Female	Eastern	Hair[a]
156	Female	Eastern	Hair[a]
147	Male	Eastern	Hair[a]
282	Female	Eastern	Blood[b]
148	Female	Central	Hair[a]
11	Female	Central	Blood[c]
150[d]	Female	Nigerian-Cameroonian	Hair[a]

[a]The genomic DNA was extracted using a QIAamp DNA Investigator Kit (QIAGEN GmbH, Hilden, Germany).

[b]The genomic DNA was extracted using a DNeasy Blood & Tissue Kit (QIAGEN GmbH).

[c]The genomic DNA was prepared by the late Dr. Osamu Takenaka (Primate Research Institute, Kyoto University) and Dr. Miho Inoue-Murayama (Wildlife Research Center of Kyoto University).

[d]The subspecies of the individual was identified only maternally due to a lack of information about the antecedents in captivity.
