# Supplementary material for: Eco-Geographical Diversification of Bitter Taste Receptor Genes (TAS2Rs) among Subspecies of Chimpanzees (Pan troglodytes)
Source: PLoS One. 2012 Aug 16;7(8):e43277. doi: 10.1371/journal.pone.0043277 (PMC3420883; doi:10.1371/journal.pone.0043277)
Supplement: Table S4 — PCR primers used in this study. These primers were designed based on CGSC 2.1.3/panTro3. (PDF) [file pone.0043277.s006.pdf]

Table S4. PCR primers used in this study. These primers were designed based on CGSC 2.1.3/panTro3.

Locus	Forward primer		Reverse primer	
	**Name**	**Sequence**	**Name**	**Sequence**
c*TAS2R9*	cTAS2R9_F	5´-TTGTGCAAATTTAAGAAATCAG-3´	cTAS2R9_R	5´-AAGGAACACAAAACAAATCTTC-3´
c*TAS2R31*	cTAS2R31_F	5´-TTGTCCTTTCTCTCTTCTCAAT-3´	cTAS2R31_R	5´-GAGCATGTTATTGTCAATGTTC-3´
c*TAS2R42*	cTAS2R42_F	5´-GCATCCCTTAGTAGATCTTTTG-3´	cTAS2R42_R	5´-CCTGGTAGGATTTCATTTATGT-3´
c*TAS2R50*	cTAS2R50_F	5´-AGAATGTTACTGCACATTCAGA-3´	cTAS2R50_R	5´-TCTATAATTTGGCATCTTGTGA-3´
IntA	intA_F	5´-GATAATTTTGTTGAGGCATAAAG-3´	intA_R	5´-TTTTCAAAGCACTAGAGTTAAGG-3´
IntB	intB_F	5´-TGTCAGATGTGTAGATTGAAAAA-3´	intB_R	5´-AACTTGTCTCTCTAGTTGGTTCA-3´
IntC	intC_F	5´-GAGGAATTATCAAGAGGAAAAAT-3´	intC_R	5´-TTTATTGTGGAGCATAACATACA-3´
Int31-63	–	–	int31-63_R	5´-GACGAGTAATAATAATTTCTATGAGATA-3´
